# Long-term demise of sub-Antarctic glaciers modulated by the Southern Hemisphere Westerlies

**DOI:** 10.1038/s41598-021-87317-5

**Published:** 2021-04-16

**Authors:** Jostein Bakke, Øyvind Paasche, Joerg M Schaefer, Axel Timmermann

**Affiliations:** 1 Bjerknes Centre for Climate Research, Allégaten 70, N-5007 Bergen, Norway; 2 Department of Earth Science, University of Bergen, Allégaten 41, N-5020 Bergen, Norway; 3 NORCE Climate, Allégaten 70, N-5007 Bergen, Norway; 4 Lamont-Doherty Earth Observatory, Columbia University, Palisades, NY 10027 USA; 5 IBS Center for Climate Physics, Institute for Basic Science, Busan, South Korea; 6 Pusan National University, Busan, South Korea

**Keywords:** Climate change, Cryospheric science, Palaeoclimate

## Abstract

The accelerated melting of ice on the Antarctic Peninsula and islands in the sub-Antarctic suggests that the cryosphere is edging towards an irreversible tipping point. How unusual is this trend of ice loss within the frame of natural variability, and to what extent can it be explained by underlying climate dynamics? Here, we present new high-resolution reconstructions of long-term changes in the extents of three glaciers on the island of South Georgia (54°S, 36°W), combining detailed analyses of glacial-derived sediments deposited in distal glacier-fed lakes and cosmogenic exposure dating of moraines. We document that the glaciers of South Georgia have gradually retracted since the Antarctic cold reversal (ACR, 14.5–12.8 ka), culminating in the disappearance of at least one of the reconstructed glaciers. The glacier retreat pattern observed in South Georgia suggests a persistent link to summer insolation at 55°S, which intensified during the period from the ACR to approximately 2 ka. It also reveals multi-decadal to centennial climate shifts superimposed on this long-term trend that have resulted in at least nine glacier readvances during the last 10.5 ka. Accompanying meridional changes in the Southern Hemisphere westerlies and their interconnection with local topography may explain these glacier readvances.

## Introduction

Glaciers and ice caps are ubiquitous along the Antarctic margin, the Antarctic Peninsula and some of the more mountainous or southerly sub-Antarctic islands; even so, most of these glaciers are in a state of decline. Since the early 1990s, the rate of ice mass loss has accelerated in West Antarctica^1^, and a recent inventory reveals that mountain- and marine-terminating glaciers on the Antarctic Peninsula have been receding rapidly, regardless of size and orientation^2^. This is also the case for South Georgia, where rare historical observations of glaciers extend to the beginning of the twentieth century^3^.

**Figure 1:**
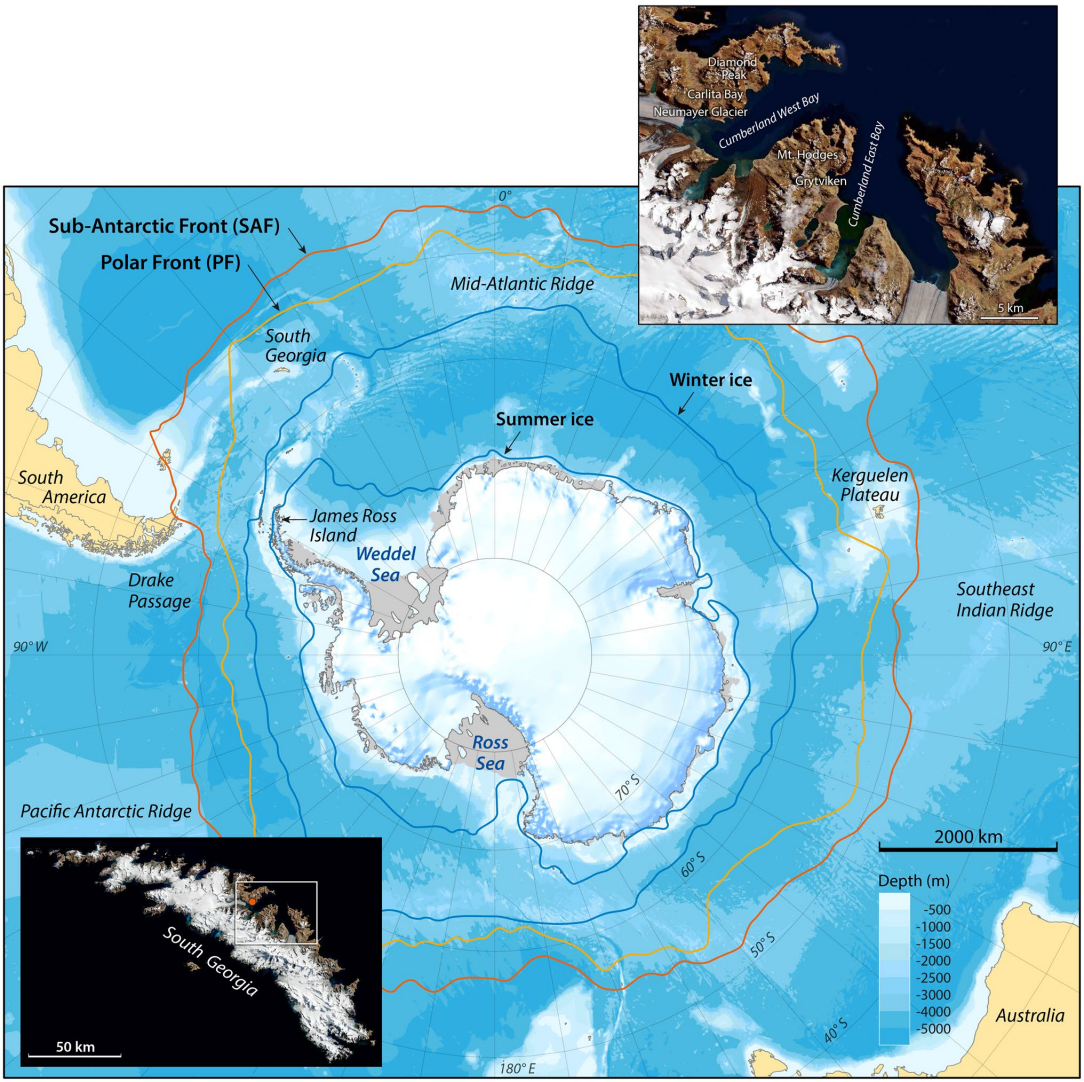
The physical setting of South Georgia in the core belt of the westerlies and between the outer limit of the winter sea ice and the polar front (fronts are based on a 30 year average, 1991–2000) (Map was created in Adobe Illustrator 2021 with data from the Australian Antarctic Data Centre http://data.aad.gov.au/aadc/mapcat/display_map.cfm?map_id=13989 and insert satellite maps are downloaded from NASA Worldview).

**Figure 2:**
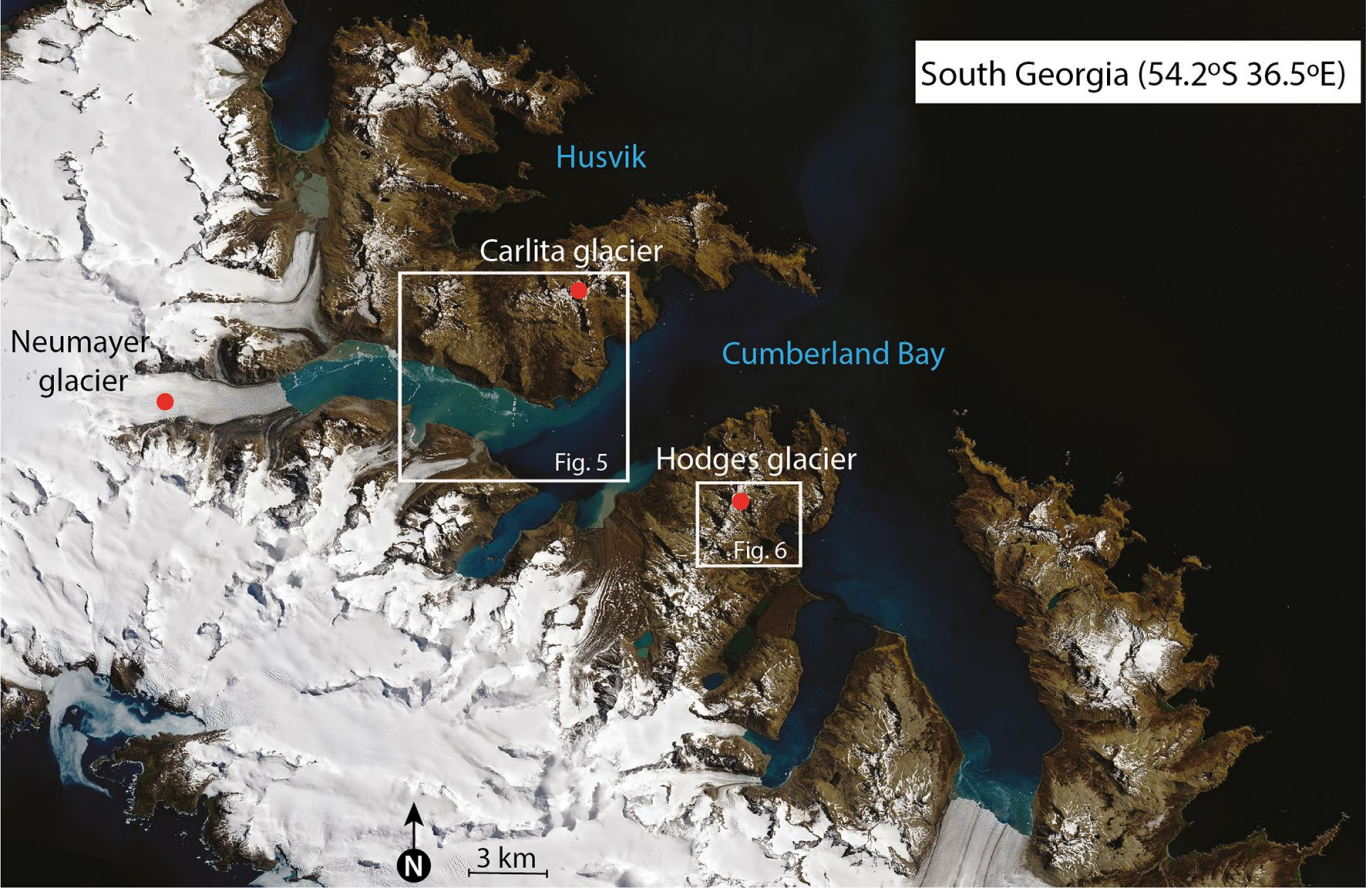
The island of South Georgia (54°S, 36°W) is 170 km long and ranges from 2 to 40 km wide; it is surrounded by a shallow shelf area with an average depth of 250 m. Glaciers and alpine mountains completely cover the central parts of the island, whereas the Cumberland Bay area is less glaciated, even though the landscape is clearly shaped by erosive glaciers, which is exemplified by the numerous cirques, u-shaped valleys, fjords, and lakes (The figure was created in Adobe Illustrator 2021 using data from NASA Worldview).

**Figure 3:**
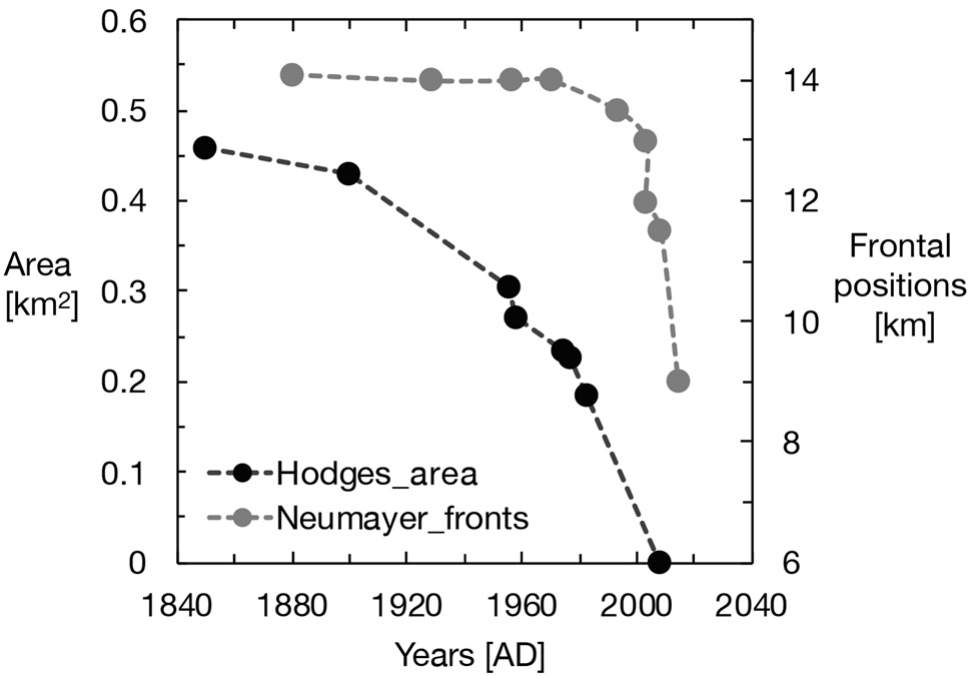
The modern retreat pattern of Hodges Glacier (primary y-axis, change measured in km^2^) in West Cumberland Bay and the larger Neumayer tidewater glacier (secondary y-axis, change measured in km extent) in East Cumberland Bay since 1850 AD. The glacier retreat patterns reveal an accelerated recession since the 1970s. The Hodges glaciers had disappeared by 2008, as observed by the authors. Neumayer is soon to become land-locked (the data for this figure are from papers^2,3,21^ and from AGU Blogshpere (https://blogs.agu.org/fromaglaciersperspective/category/south-georgia-glacier-retreat/)).

South Georgia is located c. 2200 km from Antarctica and c. 1800 km from the American continent. It lies at the edge of the modern limit of the maximum sea ice extent, close to the polar front (PF), as well as the Antarctic circumpolar current (ACC), and is embedded within the core belt of the Southern Hemisphere westerly winds (Figs. 1 and 2). Variations in the mean zonal state of the westerlies are strongly related to the Southern annular mode (SAM)—a zonally symmetric pattern, which is the leading mode of atmospheric variability in the Southern Hemisphere^4^. Dynamic shifts in the westerlies impact, among other things, the regional distribution of precipitation and temperature^5^, affecting the corresponding changes in glacier mass balances for South Georgia^6^. Glacier trends during the last century underscore their sensitivity to recent and historical warming^3,7^ (Fig. 3). Such sustained mass balance changes will be manifested by changes in glacier extent and glacial sediment supply to downstream distal glacier-fed lakes, which are typically positively correlated with ice velocity rates and glacier size^8,9^. These two features allow for reliable reconstructions of historical glacier variability, including regional climate transitions. Following the last glacial maximum (LGM) 21 ka ago, South Georgia warmed, causing a widespread recession of glaciers from the edge of the continental shelf to the inner parts of the fjords^10,11^. This trend was interrupted by a major readvance to the outer fjords during the Antarctic cold reversal (ACR) 14.5–13.0 ka ago, when the island transitioned to a 2–3 °C cooler climate^3,10,12^. Since the ACR, glaciers have gradually receded, except for a few observed glacier readvances^12–16^. Glacier retreats have accelerated in recent decades, especially on the eastern side of the island, in concert with rising temperatures^17,18^.

Addressing the response of sub-Antarctic glaciers in the past to these changing climate conditions can provide important clues about past natural variability and, therefore, their sensitivity to future climate change. To generate reliable reconstructions of past glacier extent and dynamics, we rely on a multi-pronged geological approach that combines data on the recessional moraine cosmogenic isotope exposure age with downstream datable sedimentary evidence of changes in sedimentation in distal glacier-fed lakes^20^.

**Figure 4:**
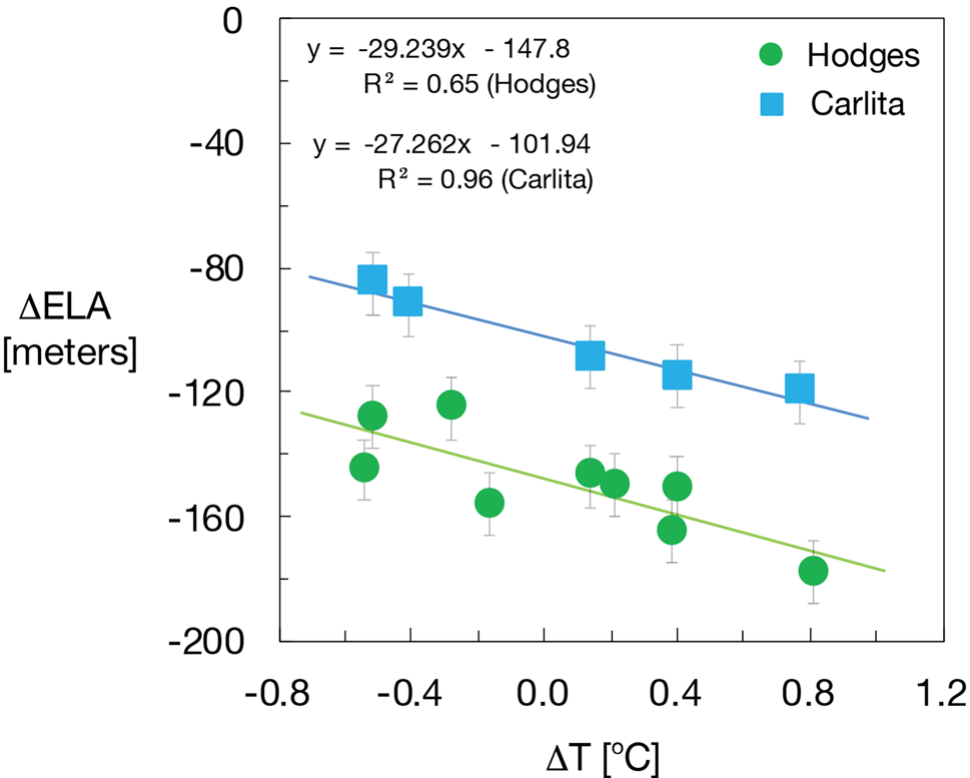
Equilibrium-line altitude (ELA) estimates based on the accumulation area ratio method for individual dated moraines in the Hodges and Carlita catchments versus summer temperature for the corresponding time interval as reconstructed at James Ross Island (see SOM for data)^25^. The trend of gradually ascending ELAs is reproduced for the two glacierized catchments. Carlita glacier is still present, contrary to Hodges, which had completely melted by 2008.

**Figure 5:**
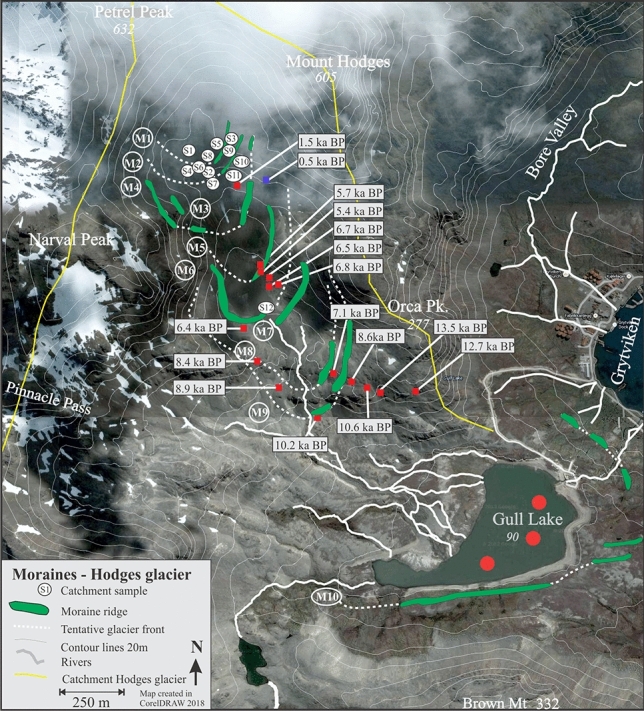
The moraines sequence in front of the Hodges glacier. Red circles indicate coring sites for the three long piston cores obtained in Gull Lake in 2008. Red squares indicate sampling locations on boulders and blue squares on bedrock surface for cosmogenic dating, and the subsequent ages are shown in white boxes (see Table S1 for details, including uncertainty estimates).

**Figure 6:**
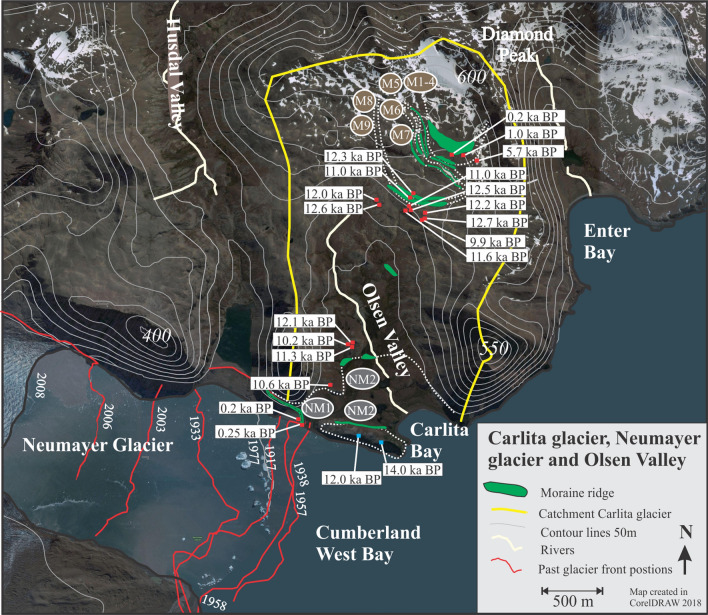
The study area in Cumberland West Bay, including the rapidly retreating glacier front of Neumayer Glacier and the much smaller Carlita Glacier lying in the uppermost part of Olsen Valley. The red lines are the historically documented frontal positions of Neumayer Glacier^44^. Red dots indicate sampling sites for cosmogenic dating, and the connecting ages are marked in white boxes.

We have carried out two extensive field campaigns in South Georgia (AD 2008 and AD 2012) to conduct sediment coring in distal glacier-fed lakes and to map glacial landforms, which allows us to constrain glacier variability. Here we present three glacier reconstructions based on a combination of historical data, dating of recessional moraines and bedrock surfaces using isotopic cosmogenic dating, as well as multi-proxy analyses of suspended sediments deposited in a distal glacier-fed lake (for details see SOM, Figs. S2–Fig. S9). Hodges Glacier is a small cirque glacier that has now completely disappeared, Carlita Glacier is a small cirque glacier in the later phases of deglaciation, and Neumayer Glacier is a large tidewater glacier that is rapidly receding (Figs. 2, 5 and 6). Together, these data provide a robust and reproducible record of past glacier activity at a decadal to centennial resolution throughout the last 14.5 k years. We use ^10^Be isotope dating in the glacier foreland to obtain an initial millennial-scale chronology (Figs. 5 and 6, Table S1). This chronology is then tested and refined by radiocarbon dating of high-resolution lake sediment records. The results presented here reveal a coherent pattern detailing several minor readvances superimposed on a sustained gradual retraction, which has culminated in accelerated retreat and thinning after AD 1950 (Figs. 3 and 4).

## Results

The exposure ages of recessional moraines mapped and sampled in two cirque glacier catchments (Figs. 5 and 6), referred to as Hodges Glacier (HG) and Carlita Glacier (CG), and one marine-terminating glacier (Neumayer Glacier, NG), were obtained using ^10^Be isotopes (Table S1). In addition, three piston sediment cores and three short gravity cores taken from Gull Lake provided sedimentary evidence that complements the Hodges recessional moraine chronology (Fig. 5). In the following, we present the results from these three complementary sites in Cumberland Bay (Figs. 5 and 6).

Hodges Glacier (HG): Before 2008, HG was located between the summits of Petrel Peak (632 m altitude) and Mount Hodges (605 m altitude) (Figs. 2 and 5). We mapped nine recessional moraines marking the former front positions of Hodges Glacier between the headwall and its distal glacier-fed lake (Fig. 5). The geomorphologic map (Fig. 5) shows the outline of the preserved moraine sequences and the sampling position for 16 ^10^Be surface-exposure dates that are used to provide a minimum age for the moraine chronology. Ages span the last 14,000 years before the present day, when the glacier is assumed to have reached the position at M10^10^ (Fig. 5). Since a glacier’s equilibrium-line altitude (ELA) can be derived from past moraine positions^21^, these positions provide a quantitative estimate of changes that must necessarily be explained by the climate at the time. The nine dated moraine ridges were used to reconstruct former ELAs using the accumulation area ratio and balance ratio methods^19^ (Figs. 5 and 6). The variations in ELA can be further used to infer temperature variations, indicating a summer warming of 1–1.5 °C (Fig. S1, see, for instance, Ohmura^20^) from the largest to the smallest glacier (Fig. 3). The youngest moraine was formed some 525 ± 50 years ago, and the reconstructed glacier had an ELA of c. 450 ± 50 m altitude. Photos presented in Gordon^21^ show the extent of the glacier in 1955 and in 1982. Mapping during the 2008 field expedition confirmed that the glacier had melted (Fig. 5).

Ground penetrating radar profiles in Gull Lake, which traps sediment-laden meltwater from Hodges Glacier, suggest that the recovered cores capture sediments (with a grain size finer than 63 μm) deposited in the lake after the deposition of M10, which is assumed to have occurred during the ACR^10,11^ (Fig. S3). This is further supported when correlating the stratigraphy, as reflected by the magnetic susceptibility (MS) measurements from the three individual cores (Fig. S4). Nineteen radiocarbon dates of macrofossils were used to establish the age–depth relationship of the lake sediment record (Figs. S8 and Fig. S9). The sediment cores from Gull Lake indicate continuous sedimentation from 10 ka until the glacier melted in AD 2008 (Fig. S8). There is therefore sufficient overlap between the radiocarbon-dated lacustrine record and the corresponding ^10^Be chronology of the nine moraines. Detailed analysis of the sediment cores included geochemical composition, density, water content, organic content, environmental magnetism and grain size (see SOM for details and Figs. S2–Fig. S9). Eleven sediment parameters were analysed using principal component analysis (PCA) (Fig. S6). The two leading principal components (PCs 1, 2) explain 64% and 13% of the total variability, respectively (Fig. S6). The parameters dry bulk density (DBD), MS, potassium (K), titanium (Ti) and calcium (Ca) show high scores along the first principal component axis, indicating that they respond to the same process or processes that take place within the catchment (Fig. S6). In order to test the degree to which the glacially produced sediments imprinted the sediment archives, a series of catchment samples were collected from the foreland between Gull Lake and Hodges Glacier (Fig. S2). The resulting analyses suggest that the glacial sediments produced, transported and subsequently deposited in Gull Lake are adequately captured by the MS and Ti concentrations (Fig. S5). The two parameters are positively correlated, reinforcing the interpretation that the lake sediment record contains an unperturbed signature of up-valley glacier activity that is detectable. In short, this means that increased glacier erosion due to increased deformation rates and larger glaciers yield sediments with higher MS values and Ti count rates (Figs. S7 and Fig. S9).

Carlita Glacier (CA): Nine recessional moraines were mapped downstream of the Carlita cirque glacier in East Cumberland Bay, south of the summit of Diamond Peak (600 m altitude) (Fig. 6) (*22*), which forms the basis of former glacier outlines and hence ELA estimates (Fig. S1). The glacier covered close to 1 km^2^ at its largest extent compared to the present day—only 0.1 km^2^ (Fig. S1)^22^. Beryllium (^10^Be) samples were collected from five of the recessional moraine systems. The exposure dates reveal that the glacier reached a maximum position during 13 ± 0.5 ka with an ELA of c. 360 ± 50 m asl. The innermost moraine system is dated to 300 ± 125 years before present (BP), suggesting an “Little Ice Age” (LIA) age of the moraine system with a corresponding ELA of 470 ± 70 m asl.

Neumayer Glacier (NG): This glacier extends to an elevation of more than 1000 m and is more than 20 km long (Fig. S1). The NG record differs from the two cirque glaciers; it has only three moraine remnants on land. A survey of the East Cumberland Bay fjord seafloor suggests that at least two recessional moraines have been deposited outside these systems, with the most proximal deposited just after 14.8 ka^11^. Ages of a bedrock ridge running parallel with the fjord suggest that NG retreated ~ 13.5 ka ago, as shown in Fig. 6. The two lateral moraines were deposited 200 years ago, and the retreat from the younger moraine took place in the early AD 1970s. No ELA estimates were calculated for NG due to the different dynamics of tidewater glaciers versus small cirque glaciers, although frontal positions are documented.

**Figure 7:**
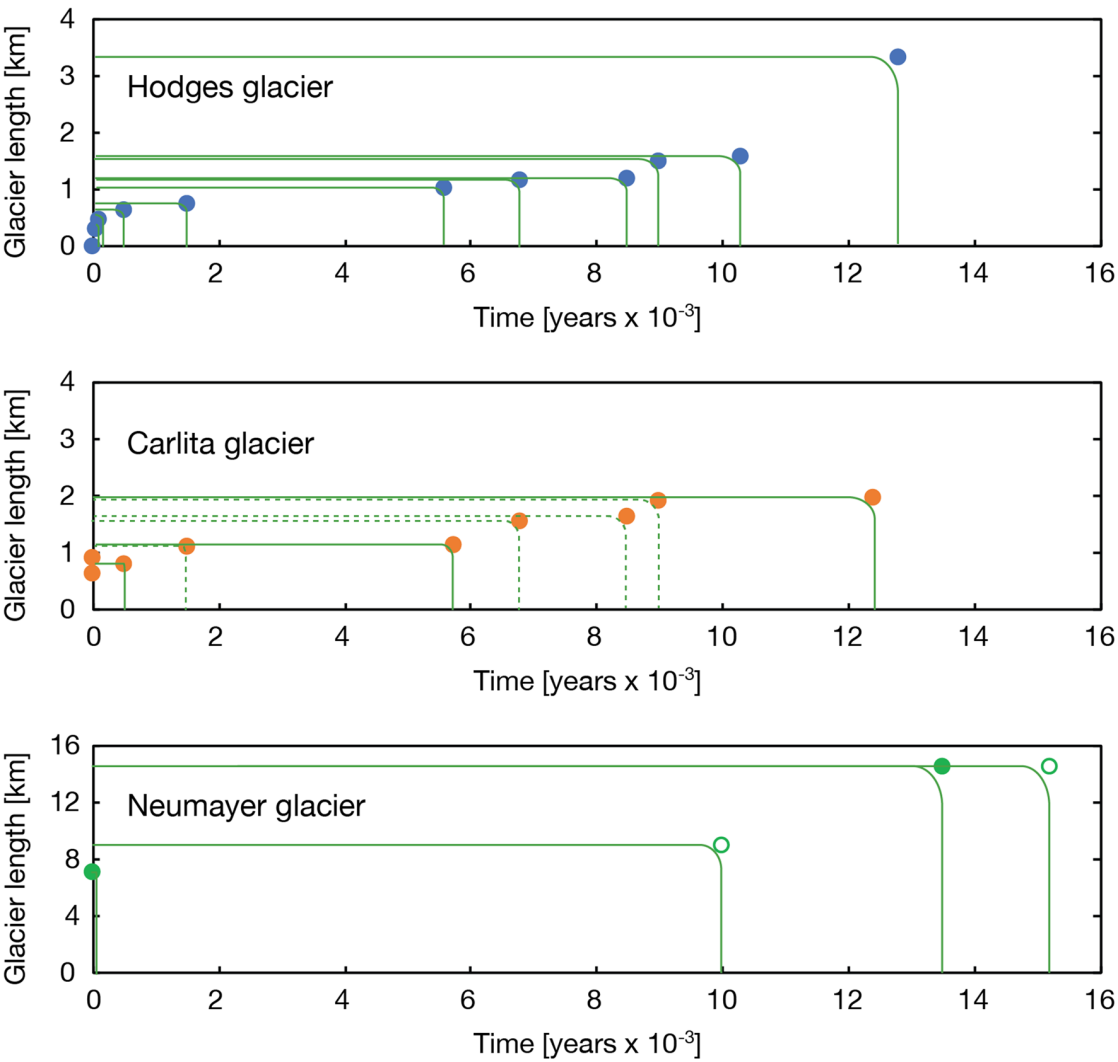
Exposure dating of glacier extents (marked by dots in the three panels representing mean age with reference to Table S1) for the three examined glaciers in South Georgia: Hodges Glacier, Carlita Glacier and Neumayer Glacier. Additional constraints on the extent of Neumayer Glacier are provided by a recently radiocarbon-dated lake sedimentary archive in the lower part of Olsen Valley^22^, denoted by an open circle at ca. 10 ka. Moreover, Graham et al.^11^ provide a limit for the horizontal extension of Neumayer Glacier during the Antarctic cold reversal (ACR) at ca 15.5 ka. Dotted lines in the upper panel are reconstructed glacier advances based on bog and lake sediments from Carlita Bay (Fig. 6)^15^.

The three individual glacier reconstructions show a common retreating trend that lends support to the idea of a response to insolation during the Holocene period^23,24^ (Fig. 6). The shared pattern of past glacier variability among our three sites reveals rapidly retreating glaciers at the end of the ACR with a decelerated retreat after ~ 10 ka. Between 10 and 5.7 ka, the glaciers retreated further, except for some minor readvances. The onset of the sub-Antarctic Neoglacial period included several glacier readvances commencing at ~ 4 ka, with at least two readvances during the last 0.5 ka (Fig. 7). There seems to be an overall rising trend for the regional ELA over Cumberland Bay throughout the entire record up to the present day, interrupted by shorter periods of temporary lowering of the ELA or stagnation of upward ELA migration accompanying periods of recessional moraine formation (Figs. 5 and 6). Based on these dates, the moraines are in stratigraphic order, meaning that none of the subsequent glacier advances is larger than the preceding advance.

## Discussion

**Figure 8:**
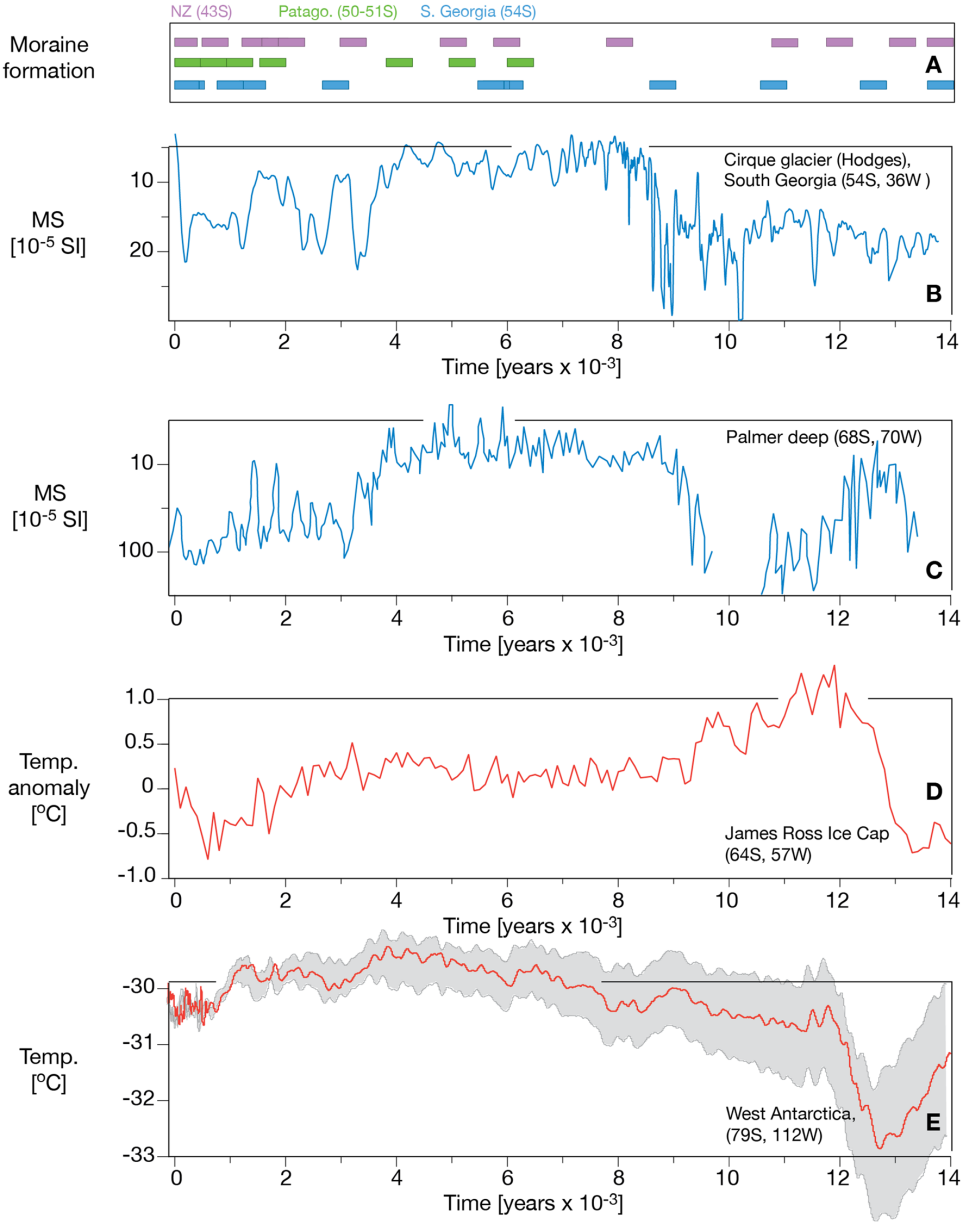
Key records of climate and glacier variability in the circumpolar fringe zone including (from above) a compilation of glacier advances for New Zealand (NZ)^28^, Patagonia in South America^26,30^ and data from South Georgia (in this paper). The second panel from the top shows the variability of Hodges Glacier based on the sediment record from Gull Lake (this paper). The third panel shows glacier variability at the Antarctic Peninsula, Palmer Deep^29^. Note that the MS values are reversed for both Hodges Glacier and Palmer Deep. The fourth panel shows the temperature based on an ice core from the James Ross Ice Cap (47), and the last panel from the top shows the temperature as recorded in West Antarctica^33^.

Our composite reconstruction from South Georgia covers the past 14.5 ka (Figs. 7 and 8). The deglaciation following the ACR started at approximately 13.2 ka and continued until it was interrupted by a cooling (reversal) beginning at 11.0 ka^25^. This resulted in recessional moraine formation around 10.5 ka and 9 ka (Figs. 5 and 6). The mid-Holocene took place from 9 until 3.4 ka, with moraine-forming glacier advances at ~ 8.8 ka, 6.7 ka, 5.7 ka and 3.4 ka (seen only in the sediment archive from Gull Lake) (Fig. 5 and Fig. S9). The three latter events coincide with the largest Holocene Glacier advances in Patagonia^26^. A drift towards a slightly cooler climate after 4 ka marks the onset of the Neoglacial period characterised by centennial-scale glacier fluctuations peaking at 3.4 ka, 2.8 ka (dated in Gull Lake) [also shown in^14^], 1.1 ka, 0.7 ka (Fig. 8), and finally, during the late twentieth century (with a maximum in AD 1910)^22,27,28^.

The new glacier records show consistency with other glacier records from elsewhere in the Southern Hemisphere, including key records from the Antarctic Peninsula, New Zealand and Patagonia^28–31^. The smoothed Palmer Deep Glacier record of meltwater inputs from the western side of the Antarctic Peninsula indicates the abrupt onset of the Holocene period at approximately 10.5 ka, followed by a quiescent phase replaced by glacier rejuvenation at 4 ka^32^, which is similar to our South Georgia records. The summer temperature reconstructed from the James Ross Ice Cap^25^ and from the West Antarctic Ice Sheet Divide (WAIS)^33^ (Fig. 8) parallels the observed long-term demise of the South Georgia glaciers, as evident from the moraine records, but it also suggests that variations in the precipitation field can help explain the multiple glacier readvances taking place on multi-decadal to centennial time scales^3,34^.

Since the termination of the ACR, December insolation at 55°S has gradually risen, eventually peaking at c. 2 ka, whereupon it weakened moderately towards the present day. Changes in summer insolation at this latitude during this time interval amount to over 30 W m^−2^^24,35^, a forcing that provides a first order explanation of the glacier trends presented here (Fig. 7 and 8).

Locally, the SAM explains 20–30% of the geopotential height variability in the circumpolar front zone and possibly more on longer time scales. This influence is not constrained to summer but includes winter as well, with a strong zonally asymmetric variability^36^. L’Heureux and Thompson^37^ demonstrated that shifts in the SAM can be partly linked to variations in the El Niño-Southern Oscillation, explaining 25% of the variance in modern data. Strengthening and poleward migration of the SAM can lead to warming in some areas of the Southern Hemisphere^7^. The Coupled Model Intercomparison Project (CMIP5) model simulations suggest that this trend is likely to continue into the next century in response to increasing greenhouse gas concentrations^38^. However, a reduction in stratospheric ozone concentrations may slow these trends^39,40^. With respect to glacier mass balance budgets, it is important to note that the SAM influences surface air temperature (SAT) in the sub-Antarctic fringe zone, not only for a specific season, but throughout the year. Specifically, Marshall and Thompson^4^ highlight that a positive SAM will warm the Antarctic Peninsula, whereas West and East Antarctic will cool, as reproduced by daily SAT anomaly data from ERA-Interim as well as station-based observations across all seasons. Although Marshal et. al’s study^41^ is based on daily data, the authors maintain that they should also be relevant on longer time scales. One local topoclimatic factor that, according to Bannister and King^37^ may amplify the climate effect of stronger westerlies are warm Föhn winds, which are prevalent in South Georgia, occurring on average 7.2 times per month. The repeated dry and warm winds on the east side of the island help to explain the asymmetrical retreat of the glaciers^3^, and are also consistent with the long-term trend of stronger westerly winds.

The glaciers of SG have retreated in accordance with increased summer temperatures in the last 100 years, concordant with the positive polarity of the SAM. We find it plausible that modulations in the SAM on decadal and longer time scales will continue to impact glacier size and extent in South Georgia, and that long-term adjustments in astral summer insolation at a latitude of 55°S can potentially be linked to a long-term southward shift in the mean position and strength of the westerlies^42^.

We speculate that changes in the SAM on decadal time scales, which by definition must be part of the natural variability of this system and allow glaciers to achieve minor advances, can be related to corresponding changes in sea ice cover, ocean circulation and possibly tropical teleconnections to the El Niño Southern Oscillation (ENSO)^36^. Further work, especially better spatial coverage of paleoclimatic reconstructions from this region, is required to disentangle the relative importance that these components have in the evolution of glacier variations documented here.

The new results detailing reproduceable sub-Antarctic glacier variability presented here reveal a trend of gradually smaller glaciers that can be traced back to the Antarctic cold reversal (ACR, 14.5–13.0 ka). Since the late onset of the Holocene period at 10.5 ka, nine glacial readvances have been recorded and dated for South Georgia. During this period, the equilibrium-line altitudes rose gradually, except for temporary stagnations associated with the formation of the moraines due to glacier readvances. Since the 1970s, glacier retreat and thinning have accelerated, and some of the smaller cirque glaciers have now completely melted for the first time in the last 14.5 ka, as evident from our lake sediment records. We speculate that the consistent shifts in the SAM, and the underlying correlation with the SAT field, contribute to sub-Antarctic glacier variability on decadal to multi-decadal time scales, potentially with corresponding shifts in the precipitation field and interactions with local topoclimatic phenomena, such as Föhn winds. A continued warming of the sub-Antarctic following the positive trend of the SAM and strengthening of the Southern Hemisphere westerly winds could lead to an unparalleled decline of the remaining land-based glaciers, as took place in the Arctic during the Holocene thermal maximum (between 9 and 4 ka) in the Northern Hemisphere^43^.

## Supplementary Information

Supplementary Information.

## Data Availability

Data are available through the Bjerknes Centre for Climate Research’s data centre; https://www.bcdc.no and NOAA.
