# Supplementary material for: Long-term demise of sub-Antarctic glaciers modulated by the Southern Hemisphere Westerlies
Source: Sci Rep. 2021 Apr 16;11:8361. doi: 10.1038/s41598-021-87317-5 (PMC8052370; doi:10.1038/s41598-021-87317-5)
Supplement: Supplementary file 1 — Supplementary Information. [file 41598_2021_87317_MOESM1_ESM.docx]

Supplementary Information for:

**Long-term demise of Sub-Antarctic glaciers modulated by the Southern Hemisphere Westerlies**

Jostein Bakke, Øyvind Paasche, Joerg Schaefer and Axel Timmermann

The supplementary information includes additional information about the sites studied, the fieldwork, the lake survey and coring, sediment analyses, age depth modelling of lake record, cosmogenic dating, equilibrium line altitude reconstructions.

1. **Site description and previous work in South Georgia**

*Topography and physical setting*

The island of South Georgia (54^o^ S, 36^o^ W) in the South Atlantic Ocean is approximately 170 km long and ranges from 2 to 40 km wide. The longest axis is oriented northwest to southeast, and the topography is dominated by fjords, bays, glaciers and alpine mountains, including the highest summit on the island, Mt. Paget (2960 m asl.). In general, the northeast coastline is more incised by fjords and valleys occupied by glaciers than the south-west facing coast. The largest glaciers descend from the high mountain areas central on the island and flow eastward into Cumberland West Bay and Cumberland East Bay. Cumberland East Bay is an 18 km long, 2.5–5 km wide, and up to 270 metres deep fjord with the tidewater glaciers Nordenskjöld, Harker and Hamberg at the head of the fjord. Cumberland West Bay connects to Cumberland East Bay and continues as a through further out on the shelf. At the head of Cumberland West Bay, several large tidewater glaciers enter the fjord, such as Neumayer Glacier and Lyell Glacier. The island of South Georgia is surrounded by a shallow shelf area at an average depth of 250 metres, and it varies in width from 50 to 150 km. See the British Antarctic Survey’s webpage for a detailed map of the entire island (<https://www.bas.ac.uk/media-post/new-map-of-south-georgia-unveiled/>)

*Geology*

South Georgia is situated on the North Scotia Ridge at the convergence boundary between the South American and the Scotia plate (Stone, 1980a). The island is a fragment of the continental crust that is thought to have once joined the southern part of South America and the Antarctic Peninsula (Dalziel et al., 1975). Two main lithostratigraphic units of sedimentary rocks delineate South Georgia, and both encompass the study areas in the Cumberland Bay region. The formations are named the *Sandebutgen Formation* and the *Cumberland Bay Formation and* are composed of quartzose greywackes and volcanoclastic (tuffaceous) greywackes, respectively (Stone, 1980a; Trendall, 1953). Both greywackes are considered part of a thick sequence of turbidites deposited during the Mesozoic period, likely the Upper Jurassic and Lower Cretaceous periods (Stone, 1980b; Trendall, 1953, 1959). The high content of quartz in the bedrock lithology enabled the use of ^10^Be dating as a reliable dating method for boulders embedded in terminal moraines as well as exposed bedrock.

*Climate*

The meteorological station in South Georgia is situated at King Edward Point in Cumberland East Bay. Temperature observations date to AD 1906. During the earliest years, the measurements were maintained by the Norwegian whalers and later by the British Antarctic Survey’s staff at the research station at King Edward Point. The normal period, from 1951–1980 has a mean annual temperature of ~2 °C and an annual precipitation of 1530 mm (15 m asl.) The island’s topography acts as a barrier for the prevailing south-westerly winds, and the windward south-west coast is distinctly more ice covered than the leeward north-east. The regional equilibrium line altitude (ELA) at the north-east side of the island runs at approximately 450–600 m asl. (cf. Gordon et al., 2008), and there are numerous ice-free peninsulas between the fjords and the tidewater glaciers. At the southwest side of the island, the ELA is typically found at 300 m, resulting in a heavier glaciated coastline (Fig. 1 and 2 in this paper).

*Field mapping and previous dating of moraines*

The quaternary landforms and deposits formed by the glaciers in the study area were mapped based on aerial photographs and complementary field surveys during the summer seasons of 2008 and 2012. In the field, we collected sediment samples from the catchments, taking positions with a handheld GPS receiver (Garmin CSX60). The topographic maps of South Georgia are unfortunately not well suited for detailed mapping and positioning; therefore, we used a GPS to determine the locations of different sites and sampling locations. The average error bars on the locations are on the order of 2–30 metres. In general, the landscape of South Georgia is rich in glacial deposits from the last Ice Age and from the Late Glacial and early Holocene time periods, when the glaciers in the area were larger (Clapperton, 1990).

Earlier studies of the island of South Georgia have addressed moraine chronologies and lake archives (Bentley et al., 2007; Clapperton et al., 1989a; Clapperton, 1971a; Clapperton, 1990; Clapperton et al., 1978; Clapperton and Sugden, 1988; Clapperton et al., 1989b; Gordon, 1987; Gordon and Hansom, 1986; Gordon et al., 2008; Gordon and Timmis, 1992; Rosqvist and Schuber, 2003; Smith, 1960). Clapperton (1971b) developed a conceptual framework for the moraine systems on the island: T1, beyond the coast prior to 14,000 ^14^C BP; T2, deposited prior to 10,000 ^14^C BP; T3 and T4, were deposited during the “Little Ice Age” and are constrained to the last century or so. This framework was later revised, and periods with moraine formations are argued to have taken place: (a) prior to 14,000 ^14^C BP; (b) prior to 10,000 ^14^C BP; (c) between 5,500 and 6,500 ^14^C BP; (d) between 4,200 and 3,200 ^14^C BP; (e) close to 2,200 ^14^C BP; (f) between 1460–1700 ^14^C BP and (g) during the “Little Ice Age” from the 17^th^ to 20^th^ century (Bentley et al., 2007; Clapperton, 1990; Gordon and Timmis, 1992; Oppedal et al., 2018; Rosqvist and Schuber, 2003; Smith, 1960; Van der Bilt et al., 2017).

**2. Mapping and direct dating of moraines**

In total, we sampled and dated 40 erratics and boulders embedded in the well-preserved moraines in the three glacier systems referred to as Hodges Glacier, Carlita Glacier and Neumayer Glacier (Figs. S1–2 and Table S1) as well as three bedrock exposures (one in Hodges, two in Carlita Bay). We used a hammer and chisel to sample the uppermost <3 cm of the boulders. Wherever possible, we sampled surfaces with distinctive quartz-rich veins from boulders that were well embedded on top of the crests of the moraine ridges. For some of the boulders, we collected shallow (<3 cm) cores with a petrol-driven corer equipped with a diamond bit. The sizes of the boulders were measured, their geometries were described, and they were photographed during sampling. We measured the shielding of each sampled surface by the surrounding topography using an inclinometer for every 20 degrees. For the few tilted surfaces sampled, we measured the dip angle and its orientation with a field compass and included this in the shielding correction.

All samples were subsequently processed at the Lamont-Doherty Earth Observatory Cosmogenic Nuclide Laboratory following the standard geochemical approach for quartz preparation and beryllium extraction. The ^10^Be/^9^Be analyses were performed at the Center of Accelerator Mass Spectrometry at the Lawrence Livermore National Laboratory (LLNL). Ages were calculated using version 3 of the exposure age calculator found at https://hess.ess.washington.edu/ (wrapper: 3.0, muons: 1A, consts: 3.0.3), which implements an updated treatment of muon-based production (Balco et al., 2008; Balco, 2017). All ages were calculated using “Lm” scaling and the New Zealand production rate value (Putnam et al., 2010).

The custom-designed ion source at LLNL, in addition to the high purity of our samples, produced high ^9^Be currents, yielding high precision for the ^10^Be measurements. In each run, each sample was measured 3–5 times for five minutes; the ^9^Be currents of our samples ranged from 14.5 to 26.5 µA. All ^10^Be/^9^Be values and ^10^Be concentrations were normalised to the 07KNSTD standard. The ^10^Be/^9^Be 1σ analytical error for the boulders older than 10 000 years ranged from 1.9% to 3.6%, with an average of 2.2%. The corresponding analytical error for the very young samples ranged from 3.8% to 6.3%. The overall background corrections, including the boron-10 (10B) corrections, corrections for procedural blanks and sensitivity variations of the AMS were below 1%.

*Moraine sequences in front of Mount Hodges (formerly Hodges Glacier)*

A cirque glacier was present between the four summits, Narval Peak (630 m asl.), Petrel Peak (632 m asl.), Mount Hodges (605 m asl.) and Orca Peak (277 m asl.), until it melted away towards the end of the 20^th^ century, as observed by the authors in 2008. In the glacier foreland of the empty cirque at Mt. Hodges and down to the fjord, down valley to and beyond Gull Lake, we mapped ten discontinuous moraine ridge sequences (M1–M10) that we have used to reconstruct the former size of Hodges Glacier and hence the corresponding ELA using an accumulation area ratio approach with values of 0.6 ± 0.05 (Porter, 1975). Based on the ELA reconstruction (see section 2), the moraines are grouped into four sub-sequences: (i) the outermost moraine (M10) sequence down valley from Gull Lake facing the fjord; (ii) the outermost moraine sequence up valley from Gull Lake (M9–M8) with two ridges preserved in the northern side of the valley; (iii) the intermediate moraine sequence (M7–M5) with two ridges preserved at both sides of the valley; (iv) the innermost moraine sequence with four moraine ridges (M4–M1) preserved at both sides of the valley.

1. *The outermost moraine sequence down valley of Gull Lake:* The outermost moraine (M10) is partly preserved, and only a few sections of the moraine can be mapped. However, the frontal position of the glacier can be spotted on satellite images by identifying sediment accumulations down valley of Gull Lake. On the east slope of Gull Lake towards the fjord, 10–20 m thick cones of partially preserved diamicton are observed. They represent the delineation of the outermost moraine deposited on land at a time period when Hodges Glacier covered Gull Lake. Along the southern shore of Gull Lake, we mapped a weathered and eroded lateral moraine located approximately 40 m above the present-day lake level of Gull Lake. This moraine is interpreted as being time synchronous with the deposit in front of Gull Lake. The moraine ridge M10 is not directly dated, but the minimum age is based on three ^10^Be ages of erratics (HOD-3, 12.7 ± 2 ka BP; HOD-4, 13.5 ± 0.8; and HOD-5,10.6 ± 0.2) taken outside moraine M9. The mean age was 13.2 ka BP, which is most likely a minimum age.
2. *The outermost moraine sequence up valley from Gull Lake:* The section consists of two moraines situated approximately 100 m apart from each other. The outermost (M9, is mapped in two sections, both at the north side of the valley. The cross section of the moraine is symmetrical, and the surface rocks look weathered. The shape and position of the moraine reflect a past glacier terminus. The moraine M8 is mapped in one section and has the same curved shape as moraine M9. On the southern side of the valley, the slope is steeper, and the terminal moraine is eroded and erased by fluvial processes and mass movement acting on the steep slope. The age of moraine M8 was based on the samples HOD-6 (7.1 ± 0.1 ka BP), HOD-15 (8.4 ± 0.2 ka BP) and HOD-16 (8.9 ± 0.2 ka BP), all sampled at the top of the moraine ridge using individual boulders embedded in the moraine. The combined mean age based on the three samples was 8.9 ± 0.2 ka BP. The age of the moraine M9 was based on two dates for embedded boulders HOD-1 (10.2 ± 0.2 ka BP) and HOD-2 (8.6 ± 0.2 ka BP).
3. *The intermediate moraine sequence:* Inboard of the outermost moraine sequence, the valley flattens where a moraine sequence has been deposited (referred to here as intermediate). It consists of three continuous elongate ridges (M7-M5). The valley floor is covered with glaciofluvial sediments deposited by the river from Hodges Glacier and on the south side of the valley, the moraines are partly buried by glaciofluvial sediments. The moraine sequence was dated using a total of six samples that were all collected from the top of the moraine ridges, so-called embedded boulders. The moraine ridge M7 was aged using one sample (HOD-14), resulting in an age of 6.4 ± 0.15 ka BP. The next moraine, M6, was dated using three samples: HOD-7 (6.7 ± 0.1 ka BP), HOD-8 (6.5 ± 0.1 ka BP) and HOD-9 (6.8 ± 0.2 ka BP). Moraine M5 was dated using two samples: HOD-10 (5.4 ± 0.2 ka BP) and HOD-11 (5.7 ± 0.1 ka BP). Ages from all three ridges overlap within one sigma, which may suggest that they were deposited relatively rapidly.
4. *The innermost moraine sequence:* The innermost moraine sequence is located 400 metres from the intermediate sequence at the top of a steep slope upwards from the relatively flat valley floor where M7–M5 were deposited. The outermost ridge is located at the brink towards the lower valley and is best preserved along the northern slope of the valley. Both moraines (M2 and M1) look fresh, with sharp edges and no vegetation. They contain boulders with a maximum diameter of one metre and are embedded in a matrix of predominantly fine silt and clay. The innermost moraine sequence *(M4–M1)* was dated using only one ^10^Be sample collected between M4 and M3. The moraine sequence was difficult to sample, as the boulder sizes were mostly under 20 cm in diameter and were generally not well suited for sampling. The sample HOD-13 has an age of 1.5 ± 0.5 ka BP, indicating that the M3 moraine is older than the “Little Ice Age” (LIA). Based on other studies from South Georgia, including our own from Carlita Glacier and Neumayer Glacier, we interpret the moraines M1 and M2 as deposited during the LIA. We discarded one sample (HOD-12; 0.5 ± 0.2 ka BP) outside M4 due to its placement close to the slope and the small size of the boulder.

*Recent retreat and melting of the Hodges Glacier*

During the last century, Hodges Glacier has diminished to half the area and length of its late 19^th^ century extent (Gordon et al., 2008). The final retreat took place during the last 30 years, and it has shrunk into two steep ice patches that can no longer be defined as glaciers (Gordon et al., 2008). The rapid retreat indicates a sustained negative mass balance on average, and it appears that the local ELA has risen above the headwall of the cirque in question. Hodges Glacier, to our knowledge, is the first glacier that has completely melted away in recent times (by 2008). During the last two centuries, the glacier has retreated approximately 900 metres (Gordon et al., 2008).

***Moraine sequences in front of Carlita Glacier***

At the south side of the summit in West Cumberland Bay, Diamond Peak (600 m asl.), a small south-facing cirque glacier (hereafter called Carlita Glacier) is located, which is less than 0.1 km^2^ in size (Fig. S2). There are no previous historical observations or field studies from this glacier or the moraines fronting Carlita Glacier, apart from those recently described in Oppedal et al. (2018). Briefly summarised, the glacier foreland contains nine moraine ridge sequences (M1–M9) that we mapped and subsequently employed to reconstruct the former sizes of Carlita Glacier. From this we estimated the corresponding ELA changes. The moraines are grouped in four different sub-sequences: (i) the outermost moraine sequence (M9–M8) consisting of two more or less continuous moraine ridges located at the brink facing towards the Olsen Valley and the fjord in Carlita Bay; (ii) the first intermediate moraine sequence in a slowly descending part of the valley (M7–M6) with two ridges preserved; (iii) the second intermediate moraine sequence (M5–M4) with two ridges preserved at both sides of the valley; (iv) the innermost moraine sequence with four moraine ridges (M3–M1) preserved at both sides of the valley close to the glacier front.

1. *The outermost moraine sequence:* Two distinct moraine ridges, M9 and M8, are deposited at the knickpoint (at an altitude of approximately 170 m), separating the upper glacier foreland from the lower Olsen Valley. The region adjacent to where M9 and M8 run is nearly devoid of superficial sediments, and there is exposed bedrock on both sides of the rivers that cuts through the moraines (Fig. S2). Both moraines are relatively intact and clearly outline the shape of the former glaciers. Cross-sections of the moraines show an asymmetric profile with a steeper slope towards the valley. The proximal side has a low angle of approximately 10^°^whereas the distal side is close to 30^°^. The two moraines are separated by 40 metres. The ages of M9 and M8 are constrained by seven samples from boulders outside the moraines (CA-01: 12.0 ± 0.2 ka BP; CA-02: 9.9 ± 0.4 ka BP; CA-03: 12.5 ± 0.2 ka BP; CA-04: 12.6 ± 0.2 ka BP; CA-05: 11.4 ± 0.2 ka BP; CA-06: 12.3 ± 0.3 ka BP; CA-12: 12.4 ± 0.3 ka BP; and CA-13: 11.6 ± 0.2 ka BP) and two samples taken directly in the moraine on embedded boulders M9 (CA-07; 12.3 ± 0.3 ka BP) and M8 (CA-08; 10.8.0 ± 0.2 ka BP).
2. *The mid-outer moraine sequence:* This moraine sequence (M7–M5) consists of two well-preserved moraine ridges and one partly preserved moraine ridge approximately 550 metres further up the valley from the outermost moraine system. The moraines are located at a threshold in the valley of about 230 m asl. The meltwater river enters a canyon distal from the moraine and towards the lower valley. A present-day meltwater river cuts through the moraine, and there are no other similar erosional contacts indicating that the present-day drainage pattern has been preserved in the past. It was not possible to find any suitable embedded boulders that could be sampled for cosmogenic dating, and the ages of these moraines therefore remain unknown.
3. *The mid-inner moraine sequence* is located less than 50 metres from M5 further up the valley. However, the geomorphology of the moraine system is different, with numerous small ridges deposited close to each other. The main moraine stage (M4) can be followed over 700 m. The present-day meltwater river cuts through the ridge at a single site. On the north side of M4, at least 15 saw tooth-shaped ridges that are less than one metre in height were mapped. The sediments in the moraines were less coarse than older moraines. The succession of ridges described here is marked on the map as M3. Our interpretation is that the moraine sequence represents moraines deposited by a rapidly receding glacier. The age of these moraine stages is constrained by three samples located outside the M4–M1 moraine complex of boulders situated on exposed bedrock (CA-09: 2.5 ± 0.1 ka BP; CA-10: 1 ± 0.1 ka BP; CA-11: 5.7 ± 0.1 ka BP).
4. *The innermost moraine sequence* makes up a collection of more than 30 individual moraine ridges deposited in a 200-metre-wide area at approximately 320 m altitude. The ridges are between 0.5 and 1.5 metres high and are strongly asymmetrical, with a much steeper distal face (35°) and a gentler (20°) proximal face. Our interpretation is that the entire moraine sequence represents annual moraines deposited during a recent recession phase of the glacier. The quality of the moraines is very similar to the description provided by Lukas (2012) showing annual moraines formed during temporary halts.

*The recent retreat of Carlita Glacier:* There are no historical observations or photo documentation of Carlita Glacier that we have come across. During two field campaigns in 2008 and 2012, we observed and measured the entire front of the glacier using handheld GPS units. Based on measurements taken four years apart, the glacier front has retreated approximately 150 metres.

***Moraines in front of Neumayer Glacier***

At the head of the Cumberland West Bay, three tidewater glaciers enter the fjord: the Neumayer, the Geikie and the debris-covered Lyell Glacier (Fig. 6). Neumayer Glacier is 14.4 km long from headwall to calving front and has its source area in a large valley head, where it is supplied with mass from the icefields of the Allardyce Range. A bathymetric study of the fjords of South Georgia revealed moraine deposits that constrain the glacial history of tidewater glaciers calving into fjords in South Georgia (Hodgson et al., 2014a; Hodgson et al., 2014b). In Cumberland West Bay, no evidence of former glacier front positions was found in front of Neumayer Glacier, except for some partially preserved moraine fragments north of Enter Bay (Fig. 6). However, glacial geomorphological mapping onshore in Carlita Bay and in the lower Olsen Valley revealed evidence that Neumayer Glacier indeed entered the lower part of the Olsen Valley and deposited lateral moraines (Fig. 6) (cf. Oppedal et al., 2018). At the north side of this mountain ridge, there is a prominent lateral moraine (named NM1 in Fig. S2) sloping down from 60 m asl. to 45 m asl. We suggest that the moraine ridge was formed when Neumayer Glacier advanced and entered the lower part of the Olsen Valley. Approximately 1.5 km northwards from the beach in Carlita Bay, there are several cones with superficial material that protrude above the bog that otherwise covers most of the lower valley. These cones are interpreted to represent remnants of a marginal moraine system (named NM2 in Fig. 6) deposited when Neumayer Glacier entered farther into Olsen Valley.

Presently, Neumayer Glacier is situated c. 7 km westward up the fjord from Carlita Bay. The recent retreat of the glacier is documented through historical observations and pictures taken by early explorers (e.g., Ernest Shackleton) and are shown as red lines in front of the glacier in Fig. 6 (Cook et al., 2014). Neumayer Glacier stabilised between the mid-1950s, and 1970 but then retreated more than 7 km until the present time. The minimum age of the lateral moraine named NM2 in Figure 5 is defined by several cosmogenic dates outside the moraine complex (CA-13: 11.8 ± 0.2 ka BP; CA-14: 14.0 ± 0.2 ka BP; CA-15: 11.8 ± 0.2 ka BP; CA-16: 12.3 ± 0.4 ka BP; CA-20: 11.9 ± 0.2 ka BP; CA-21: 10.1 ± 0.2 ka BP; CA-22: 11.1 ± 0.2 ka BP). The ages span 4ka years with an average of age close to 12 ka BP. This places the moraine system in the end of the Antarctic Cold Period. The lateral moraine named NM1 in Figure 5 was dated using two cosmogenic samples on embedded boulders in the terminal moraine (CA-17: 0.2 ± 0.1 ka BP; CA-18: 0.25 ± 0.1 ka BP). These ages places the moraine system in to the “Little Ice Age”.

Table S1. Cosmogenic dates from samples in front of Hodges Glacier as well as Carlita Glacier and Neumayer Glacier.

**3. Reconstruction of Equilibrium-Line Altitudes (ELAs)**

The ELAs for all former glacier positions were estimated based on reconstructed glacier outlines using a cartographic approach (Carr et al., 2010) where the mapped marginal moraines in front of the Hodges Glacier and Carlita Glacier (Table S2) were used to reconstruct the frontal positions of the paleo-glaciers. The glaciers were reconstructed using both the Accumulation-Area-Ratio (AAR) method and the Accumulation-Area-Balanced-Ratio (AABR) method. The AAR method assumes that the steady-state AAR of former glaciers is typically 0.55-0.65 (Porter, 1975). An AAR of 0.6±0.005 is generally considered to characterise the steady-state conditions of cirque glaciers. The AABR method is a refinement of the AAR approach, in which variations in the glacier’s hypsometry are considered making it more suitable for Valley Glaciers. Since accumulation and ablation gradients are controlled by different climatic variables, the accumulation and ablation gradients generally have different values, with the ablation gradient somewhat steeper than the accumulation gradient. Rea (2009) provided an empirically derived dataset characterising AABR ratios, which may be used for ELA estimation based on a worldwide distributed data set. We tested different gradients ranging from 1.0 to 3.5; however, it is likely that the glaciers of South Georgia have a rather high mass balance gradient due to the maritime climate.

Table S2. Reconstructed ELA changes for Hodges Glacier

Table S3. Reconstructed ELA changes at Carlita Glacier.

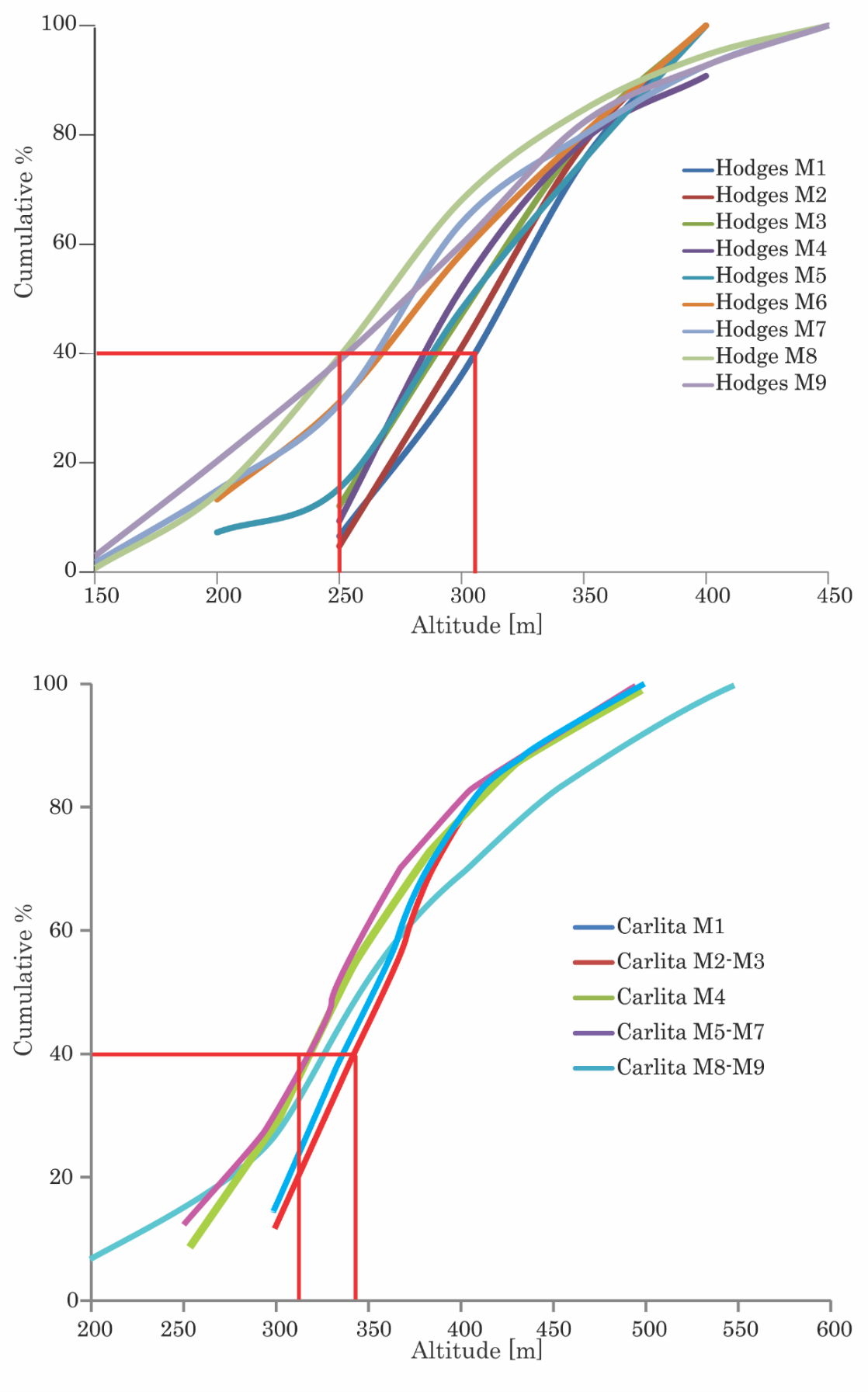


Figure S1. Cumulative area (%) distribution for the reconstructed glaciers based on the moraine sequences in the glacier foreland of Carlita Glacier and Hodges Glacier. Red lines connected to the axis show the spread of ELA scenarios when the AAR is set to 0.6.

*Catchment samples for Hodges Glacier*

Twenty-four sedimentary catchment samples were collected from (i) the main glaciofluvial deltas of the lake, (ii) secondary stream inlets entering Gull Lake and (iii) moraine ridges in the glacier foreland of Hodges Glacier (Fig. 5). Geochemical elements and magnetic properties were measured on the < 125 µm fraction. The purpose was to secure a reliable comparison with the lake sediment record from Gull Lake, given that the coarser fractions were not represented in the basin. X-ray Fluorescence (XRF) scanning were performed with the same settings as the sediment cores on the ITRAX scanner. Bulk magnetic susceptibility (χbulk) was measured at 77 K (liquid nitrogen) and 293 K (room temperature) on an MFK1 Kappabridge to estimate the relative contribution of para versus ferromagnetic minerals to the total MS. In general, the results for the catchment samples taken from terminal moraines indicate that the MS-ratio decreases up-valley towards the headwall of the cirque (Fig. S2). This shows that the relative contribution of paramagnetic material decreases as glacial material becomes fresher (younger). Based on these observations, it seems plausible to assume that glacially produced sediments will result in higher values of both MS and Ti, as recorded in Gull Lake. This interpretation aligns well with the results of the core analysis, as presented below.


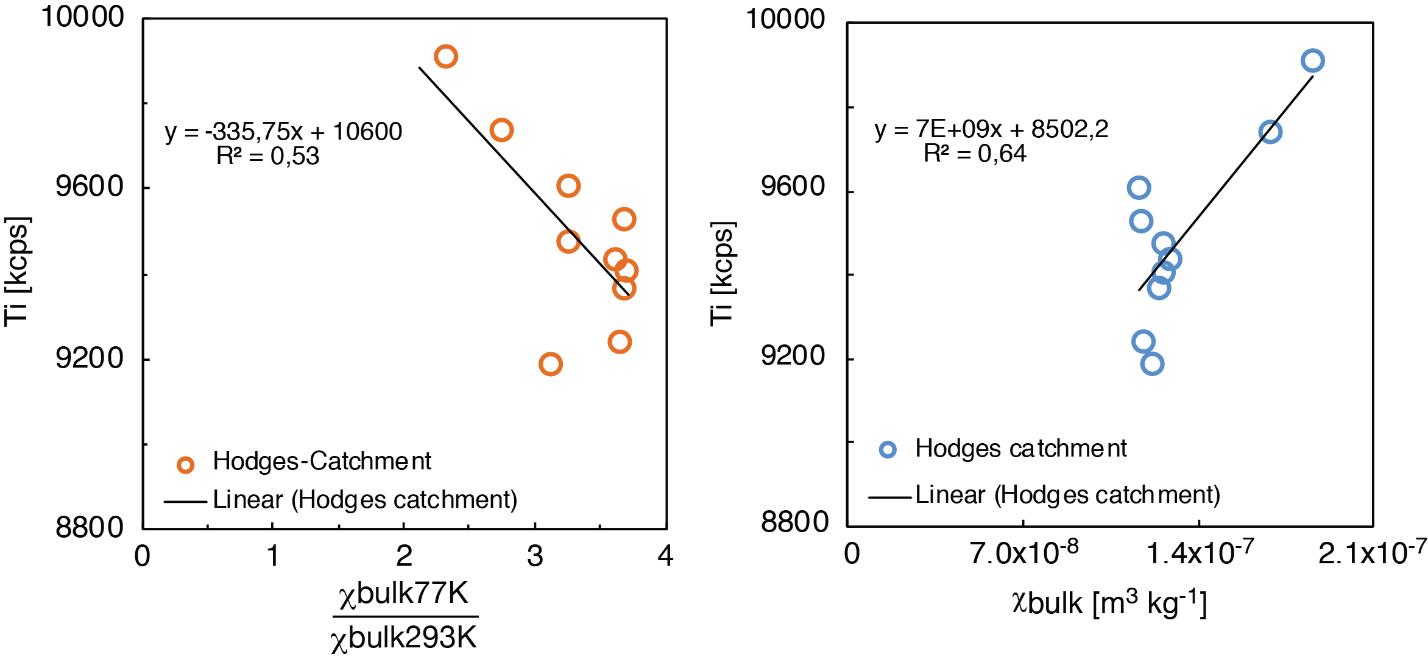


Figure S2. Eleven catchment samples were collected from the Hodge Glacier’s moraine sequence. **Left:** the figure shows the relationship between the Ti and MS ratios, where the latter is indicative of ferro- versus paramagnetic mineral contribution, where a value of 3.8 suggests paramagnetic dominance. The inverse relationship suggests that high Ti values covary with samples with a relatively high ferromagnetic contribution **Right:** the figure shows that the Ti–𝓍bulk values increase towards the head of the cirque where Hodges Glacier receded. The data suggest that the youngest catchment sediments become progressively stronger, more ferromagnetic and have a higher titanium content. Subaerial weathering and mixing with extra-glacial sediments are likely explanations for weaker values, which is in good agreement with the inverse relationship between Ti and the MS ratio presented in the figure to the left.

**4. Lake survey of Gull Lake**

Prior to coring in January 2008, Gull Lake was surveyed using a ground penetrating radar (GPR) in order to map out the distribution of soft sediments in the lake and to identify the optimal coring sites (Fig. S3). The electromagnetic pulses generated by the GPR transmitter penetrate through the soft sediments, making it possible to map both sediment thickness and the detailed bathymetry of the lake basin. For this purpose, we used a RAMAC GPR from Mäla with a 50 MHz RTA antenna inside a 10 m long PVC tube. The system has an internal differential global position system (DGPS) for positioning the profiles.

The bathymetry of Gull Lake reveals a smoothed lake bottom with its deepest part central in the lake, with a maximum water depth of 11 metres. The soft sediments are evenly distributed across the lake bed but vary in thickness from three to six metres. The coring sites were selected based on the overall sediment thickness mapped with the GPR. We note that the maximum sediment thickness was found in smaller depressions in the firm bottom. Based on our interpretation of the GPR profiles, the bedrock is overlain with a consolidated bottom till that is covered with the soft sediments that were possible to collect with our coring device, which is in accordance with the observation that Hodges Glacier advanced over the lake during the deposition of M1.


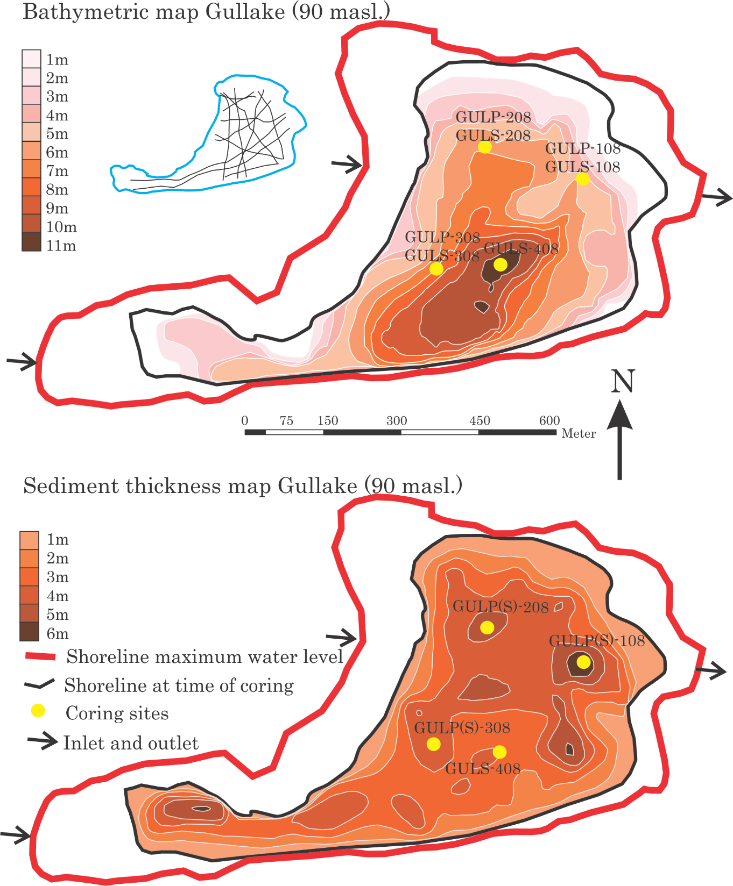
Figure S3. Bathymetric and sediment thickness maps of Gull Lake. The lake level was lowered during the fieldwork exercise due to construction work on the hydroelectric power station down the valley from the lake, and the red line marks the shoreline when the lake level is at its normal altitude. The yellow dots indicate the coring locations for the piston and gravity cores. The infill of the basin is much more complex than the bathymetry indicates, and the coring locations are based on the interpretation of the sediment thickness map.

**5. Lake coring and laboratory analyses of sediment cores from Gull Lake**

Three piston cores and four short gravity cores were retrieved from Gull Lake (GULP-108: 344.5 cm, GULP-208: 379.5 cm, GULP-308: 407 cm, GULS-108: 19 cm, GULS-208: 23 cm, GULS-308: 16 cm and GULS-408: 25.5 cm), see Fig. S3. The cores were secured from a raft using a piston corer with a 110 mm diameter core tube constructed to obtain up to 5.75 m of sediments (Nesje, 1992). The uppermost soft sediments were sampled with a small gravity corer (HTH-corer) to preserve the sediment-water interface. GULS-108, 208 and 308 were taken at the same location as the long piston cores (GULP-108, 208 and 308), and GULS-408 was taken at the deepest spot in the lake.

*Laboratory analyses*

In order to better understand and quantify the sedimentary history of Gull Lake, we performed several types of measurements on the cores described in the following. Magnetic susceptibility (MS) was measured using a Bartington MS2E sensor at 0.2 cm intervals on the three sediment cores, which were split in half and covered with polyethylene. For the short gravity cores, magnetic susceptibility was measured using a KLY-2 induction bridge (sensitivity: 4 x 10^-8^ SI) on wet samples sub-sampled in plastic bags at 0.5 cm resolution. The X-ray fluorescence (XRF) analyses were completed with an ITRAX X-ray fluorescence core scanner (ITRAX-XRF) (Croudace et al., 2006). The analysis was performed with a chromium tube, and the power was set to 30 kV and 50 mA using a counting time of 10 s at 200 mμ increments (GULP-308; n = 20067). The eight chemical elements, silicon (Si), potassium (K), calcium (Ca), titanium (Ti), iron (Fe), strontium (Sr), manganese (Mn) and zircon (Zr), have count rates above background. All elements show similar progressions, meaning high count rates in unit H (except Fe), generally decreasing count rates in units H through D, with the lowest values in unit F. Units G and E exhibit a sharp increase in count rates, whereas counts are similarly lower again in unit B. The count rates are in general agreement with the DBD values, as they are inversely related to loss on ignition (LOI) values. The samples for LOI, dry bulk density (DBD), and water content (WC), sampled continuously at 0.5 cm intervals (GULP-308; n = 825, GULP-208; n = 600), were dried overnight at 105 ^°^C in ceramic crucibles before the dry weight was measured (normally 0.5–2 g). Water content was calculated in % of total weight, and dry and wet bulk density (g/cm^3^) was measured using a syringe for fixed volume sample extraction. In the furnace, the samples were subjected to gradually rising temperatures for half an hour and then ignited at 550^°^C for one hour. The crucibles were then cooled in a desiccator for approximately half an hour and then weighed at room temperature (~18–20^o^C). The LOI weight was calculated as a percentage of the dry weight.


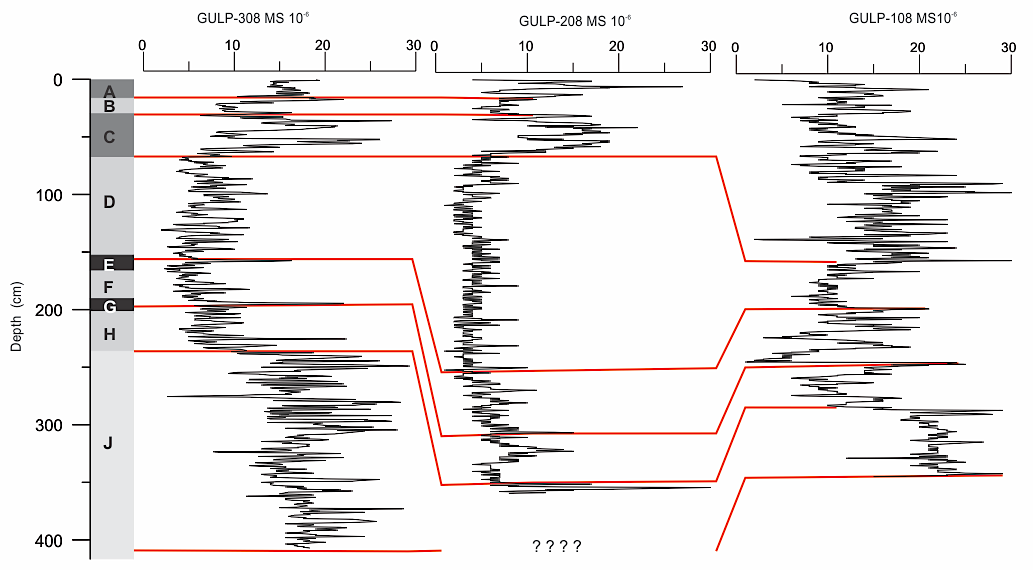


Figure S4. Correlations of the three cores based on surface MS values. Cores GULP-308 and GULP-208 are divided into nine units based on visual logging of the cores (A–J), and the lower part of GULP-108 corresponds to units J to G. The upper part of GULP-108 is probably disturbed due to sub-aquatic slumping, as the core is taken in an area of the lake with steep slopes (see Fig. S3).
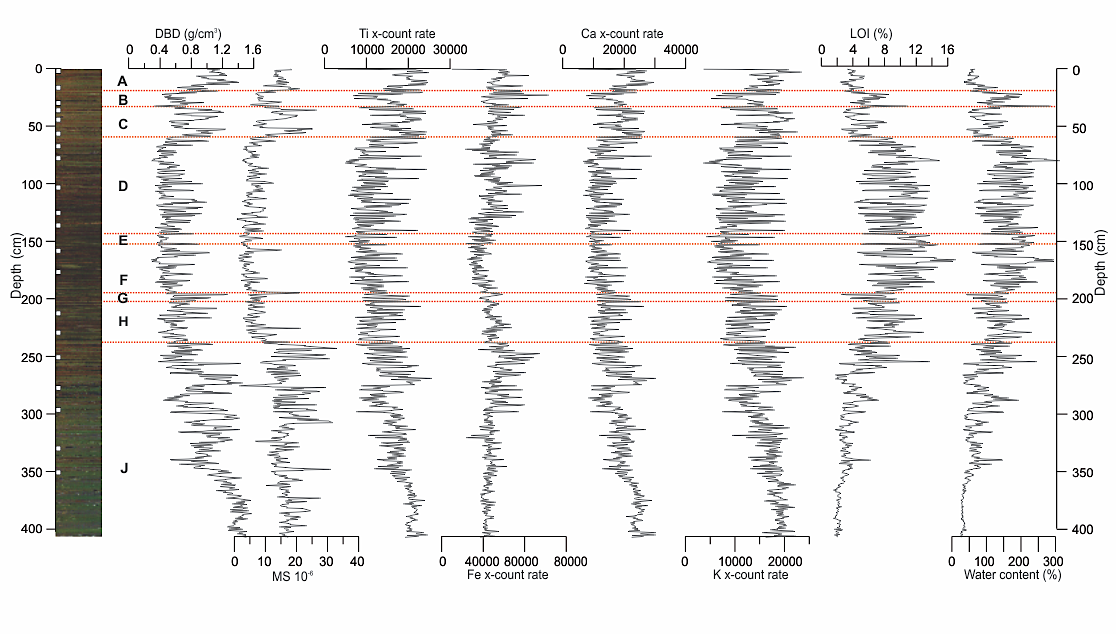


Figure S5. Sediment variables measured on core GULP-308, along with an image of the core. The samples used for radiocarbon dating are shown as white squares on the image. The unit subdivision based on magnetic stratigraphy is shown with red lines.

*Lithostratigraphy*

The lithostratigraphy in the three cores was correlated into nine units defined by visual logging and supported by MS stratigraphy mimicked by other parameters such as Fe, Ti, Si and DBD (Figs. S5–7). The cores (GULP-208 and GULP-308) contain all nine units and show that the stratigraphy of the two records is representative of the soft sediment infill of Gull Lake (Figs. S5–7). The lower part of GULP-108 contains the same units from J through F, but the uppermost part of the core deviates from the other two parts. This can be explained by the narrow steep-sided pit that appears on the sediment thickness map of Gull Lake. We used GULP-308 to describe the lithostratigraphy and define the units, as well as to provide a detailed description of the sediment parameters, as this core has the highest sedimentation rate (Fig. S8). Unit J consists of massive minerogenic sediments of clay and silt. The DBD is between 0.8 and 1.2 g/cm^3^ throughout the unit. The organic content is below 4% throughout the unit.

Altogether five radiocarbon dates are sampled from this section (Unit J: GULP-308: 407–240 cm; GULP-208: 360–250 cm and GULP-108: 350–280 cm). Unit H consists of dark brown to grey massive gyttja with layers of clay (2–8 mm thick), with DBD values between 0.3 and 1.4 g/cm^3^ and the LOI increases from 4% to 12% with high frequent fluctuations. Altogether two radiocarbon dates are sampled from this section (Unit H: GULP-308: 240–200 cm; GULP-208: 350–300 cm and GULP-108: 280–250 cm). Unit G consists of grey, silty clay grading to more organic-rich sediment at both the upper and lower boundaries (Unit G: GULP-308: 200–195 cm; GULP-208: 300–295 cm and GULP-108: 250–240 cm). Unit F consists of greyish to dark brown gyttja with many thin layers of silt/clay with DBD values varying between 0.3 g/cm^3^ and 1.2 g/cm^3^ and the LOI fluctuating between 4% and 18% (Unit F: GULP-308: 195–160 cm; GULP-208: 295–250 cm and GULP-108: 240–200 cm). Unit E consists of a grey, silty clay grading to more organic-rich sediment at both the upper and lower boundaries (very similar to unit G). The DBD values peak at 1.2 g/cm^3^ and the LOI s between 6% and 4% (Unit E: GULP-308: 160–150 cm, GULP-208: 250–245 cm and GULP-108: 200–195 cm). Unit D consists of a grey to brown silty gyttja intersected by thin laminas of silty clay, with DBD values fluctuating around 0.4 g/cm3 and LOI decreasing from 4% to 12% with high-frequency fluctuations. The laminations are separated by gradational contacts and are irregular in thickness (0.1–0.6 cm) (Unit D: GULP-308: 150–70 cm, GULP-208: 245–70 cm and GULP-108: 195–150 cm). Unit C consists of massive grey minerogenic silty clay with visible layers and laminas of clay/silt and with a graded transition into unit B; DBD has values from approximately 0.4 to 1.6 g/cm^3^ and LOI varies from 12 to 4% (Unit C: GULP-308: 70–40 cm; GULP-208: 70–40 cm and GULP-108: not possible to identify).

Unit B consists of brown to grey silty gyttja with DBD values of 0.3 and LOI values of 10%. The transitions to units above and below are graded (Unit B: GULP-308: 40–30 cm; GULP-208: 40–30 cm and GULP-108: not possible to identify). In unit A, sediments are grey, massive and have graded transitions from browner sediments in unit B, intersected by layers of light grey silt visible in lithostratigraphy. The DBD values increase from 0.9 to 1.6 g/cm^3^, and the LOI values are on average 6%.

*PCA of lacustrine sediment parameters*

Eleven parameters from core GULP-308 were analysed using PCA. The analyses include physical sediment parameters, such as MS, DBD and LOI, and geochemical sediment parameters, such as Fe, Mn, Zr, Sr, Si, Ca, Ti and K. All are commonly linked to detrital input in lake sediment records (Croudace et al., 2006; Wittmeier at al., 2015). The PCA returned an explanatory power for the first principal component of 64% of the total variability, and the second principal component had an explanatory power of 13%. The parameters DBD, MS, K and Ti (Ca) show high scores along the first principal component axis (PC-axis 1), indicating that they are responding to the same sedimentation-driven process or processes in the catchment. The main source of minerogenic sediments in Gull Lake is the sediment delivered by glacier erosion to the lake system. However, there will always be noise in the form of other catchment processes in such alpine environments, and it can therefore be difficult to distinguish the sediments produced by glacier erosion from other catchment processes. To overcome this problem, we collected samples from the catchment and compared their geochemical signatures with the lake record. It was apparent from the dilution of the Ti signal downslope from Hodges Glacier that lighter geochemical elements, such as Ti, are related to glacial sediments. PC-axis 1 and the parameters that show high scores along it, such as MS, DBD and Ti, are therefore probably the best indicators of glacier activity in Gull Lake. Based on this, we chose to use the MS record as a surrogate for up-valley glacier activity. This parameter is widely used in this context; when glacier activity is higher and minerogenic content increases, the relative amount of organic material in the core record decreases. The DBD and MS records are commonly inversely correlated with LOI.


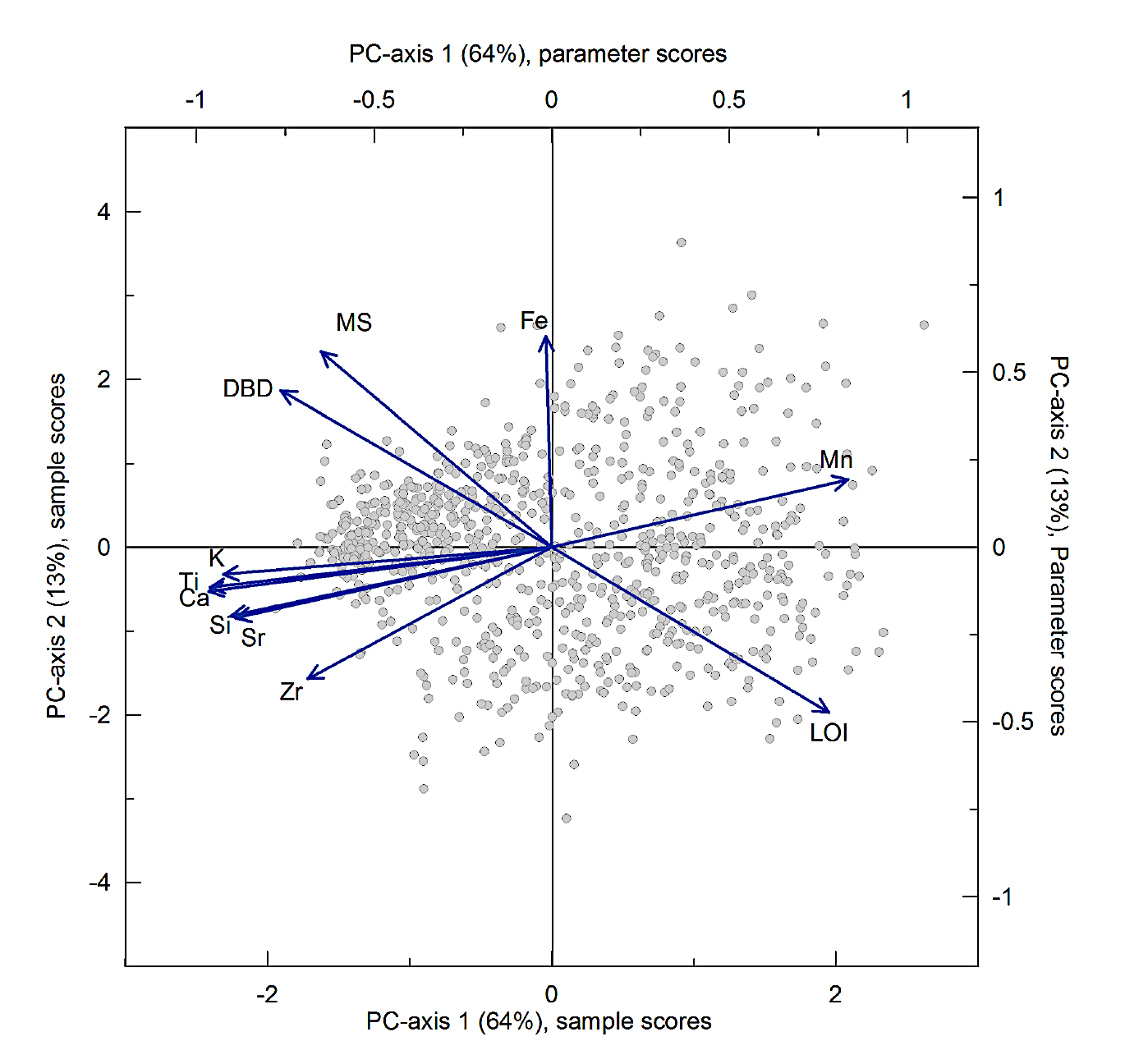


Figure S6. The PCA analyses of the multi-method sedimentary analyses in core GULP-308. Typical detrital parameters are assembled (including Ti, Si, DBD, MS, Ca, Si and K) along PCA axis 1.


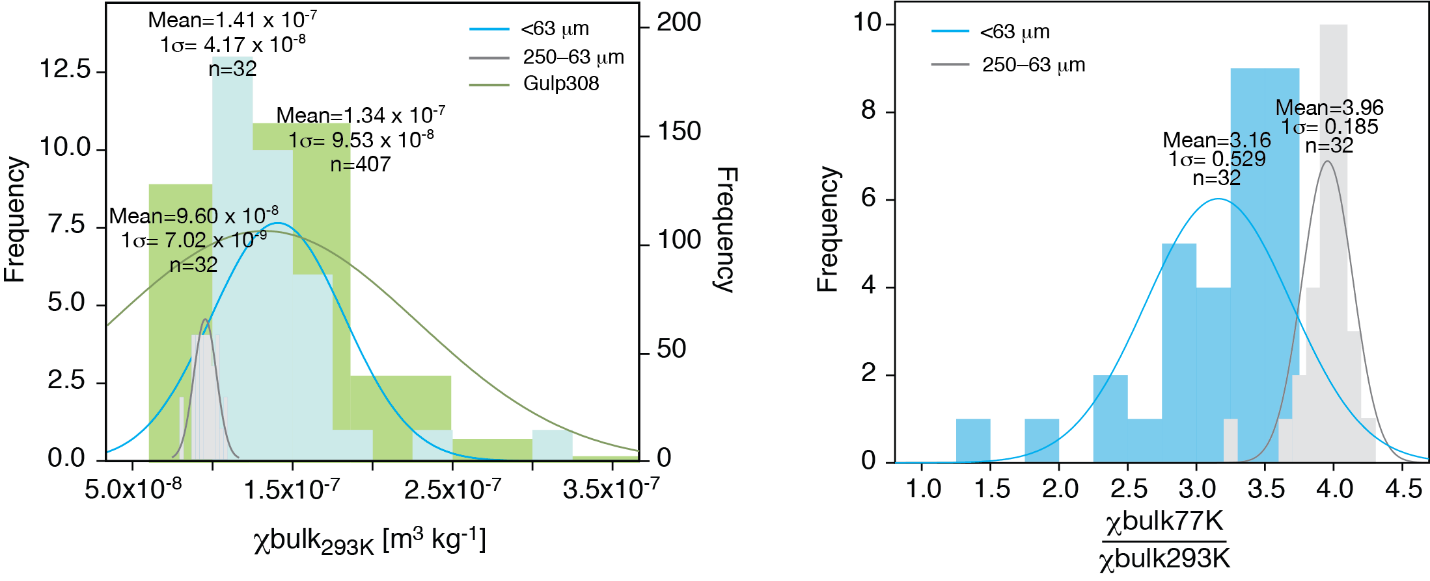


Figure S7. **Left:** Magnetic catchment sediment signatures for two bulk particle size fractions (250 mm and <63 µm) versus lake sediments that are finer than 63 µm. There is overlap between the finest fractions in the catchment and in the lake, whereas the coarser fraction is weaker and less representative of the lake sediments. **Right:** The MS ratio for the same two fractions as in the figure to the left. The ratio indicates that there is a greater content of paramagnetic minerals in the coarser fraction compared to the finer particles.

**6. Age-depth modelling**

For radiocarbon dating, plant macrofossil fragments were wet sieved (125 μm mesh) from the core GULP-308. Nineteen samples of macrofossils were analysed using accelerator mass spectrometry (AMS). Radiocarbon dating was carried out by the Poznan Radiocarbon Laboratory in Poland. Age-depth models were constructed using R-source code provided by Blaauw, the results are shown in Fig, S8. Sufficient organic material could not be extracted for AMS radiocarbon dating from the lowermost part of the core, and we therefore used the age inferred from the up-valley cosmogenic dating to define the onset of sedimentation in Gull Lake. Based on a correlation between the three short gravity and piston cores, it appears that 10 cm of the uppermost sediments is missing in the piston core GULP-308. Six of the radiocarbon dates were excluded from the age-depth modelling, as the ages were inverted. The inverted ages are explained by in wash of old organic material from the catchment. For each radiocarbon date, the actual year used for age-depth modelling was drawn based on the calibrated radiocarbon age, with sampling probability proportional to the probability density of the calibrated radiocarbon age. As suggested by Heegaard et al. (2005) and further developed by Blaauw (2010), we included a term that considers the uncertainty of the uncalibrated radiocarbon date. A loess filter (smoothing parameter = 0.75) was then drawn through the age estimates, and the 95% confidence range was 351–2062 years (the average for the entire core was 691 years) calibration probabilities of the individual age estimates. This procedure was repeated 1000 times, and 33 models were removed due to age reversals. The age-depth model shows high accumulation rates prior to 7500 cal yr BP and a slower accumulation rate towards the present day.


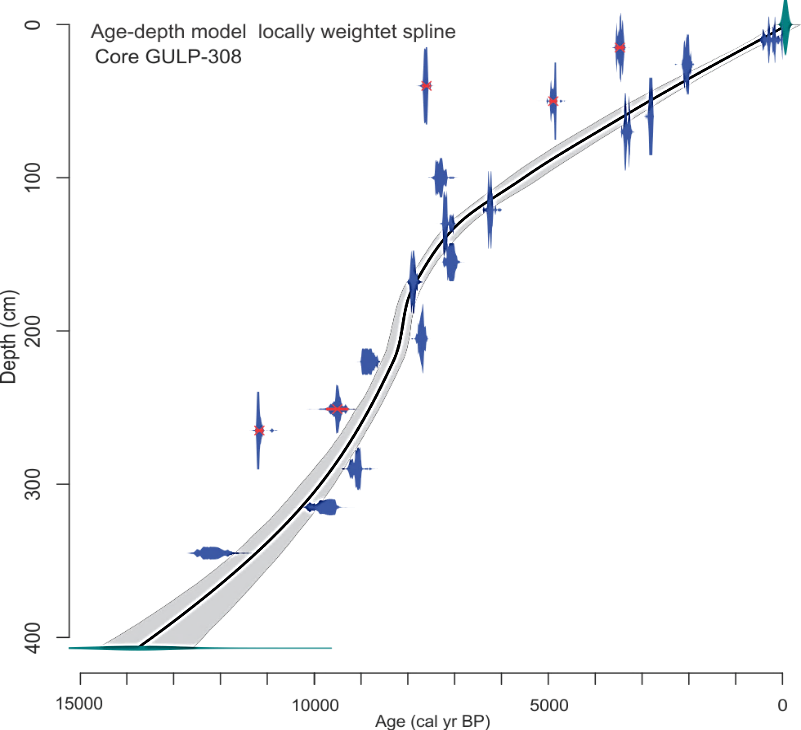
Figure S8. Age-depth modelling done in the R based script for age-depth modelling “Clam”, based on 19 radiocarbon dates. Samples with red crosses are rejected and are interpreted to represent the in-wash of older material (see Table S4).

Table S4. Radiocarbon dates on sediment core GULP-308. All samples are based on what we found in the sediments after sieving and are macrofossils of both mosses and leaf fragments.

**7. Glacier erosion and inorganic sedimentation in distal glacier-fed lakes**

Warm-based glaciers produce abundant fine-grained sediments that become entrained by meltwater during the summer season. The transport typically ends abruptly when the meltwater enters downstream lakes, where the majority of the sediment is deposited (Bakke et al., 2005a; Bakke et al., 2005b; Bakke et al., 2010; Bakke et al., 2005c; Bakke et al., 2013). The extensive analyses presented here of both the catchment and the lake sediment cores (Figs. S2 and S5) underscore that MS and Ti track past glacier history and that overall variations can be attributed to glacier size (Fig. S7 and 9). The independently dated moraines and glacier foreland lend support to this interpretation as well. During the last 10,000 years, Hodges Glacier has gradually become smaller, which increases the distance over which sediments need to be transported before they eventually become deposited. The gradual retreat throughout the last 10,000 years also exposed sediments in the growing glacier foreland that could potentially be remobilized and subsequently added to Gull Lake.

Although we cannot exclude the possibility that increased glacier–lake distance or a temporary imprint of paraglacial sediments could modify the MS and Ti lake sediment records to some extent, we suggest that both these potential sources could only have a minor influence on the multi-decadal to centennial shifts and trends in glacier variability that we observe (Fig. 9). The only possible exception to this is the relative strength of the signal after 4,000 years, which, given the modest extent of the glacier otherwise, would suggest a weaker signal in both MS and Ti compared to the early Holocene period. Another (though not necessarily competing) explanation for this is that the glacier is somewhat warmer during this later interval due to a higher mass balance turnover. The similarity in glacier extent in both space and time for Hodges and Carlita, as constrained by their dated positions, precludes that this can be attributed to glacier extent alone.


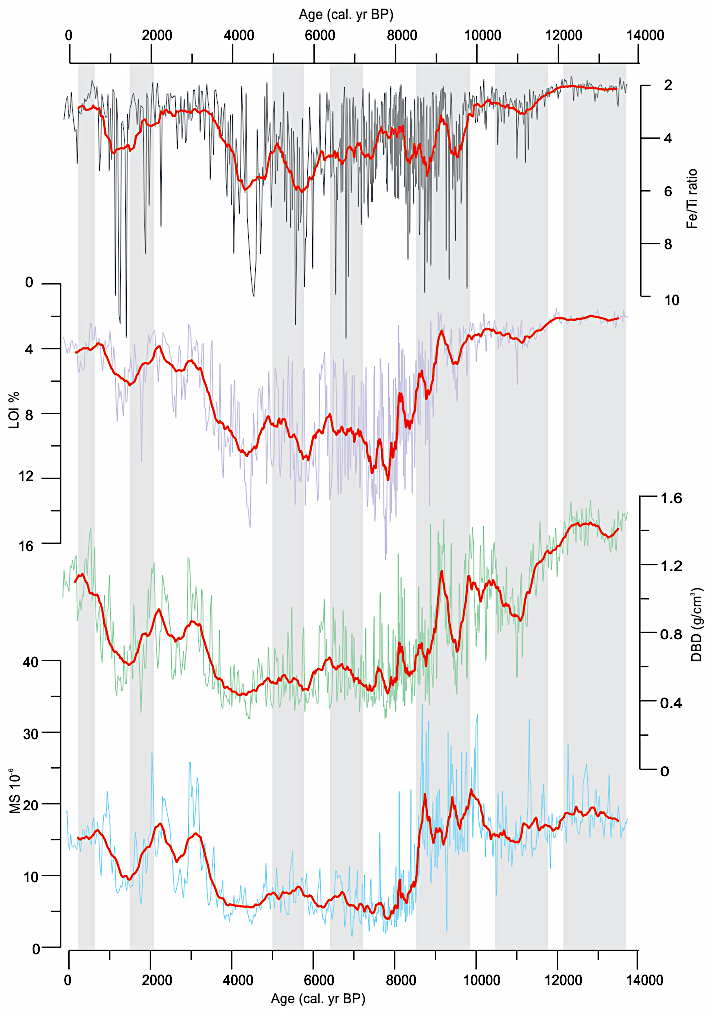


Figure S9. Selected sediment parameters reflecting the inorganic sedimentation in Gull Lake. The MS, DBD, inverse LOI, and Fe/Ti ratios show the same main pattern. The read lines are a moving average of 30 years. The grey shaded areas are periods with moraine formation in front of Hodges Glacier. Note that there are periods where the moraine formations lag the increase in inorganic sediment accumulation in Gull Lake.

1. **References**

Bakke, J., Dahl, S.O., Nesje, A., 2005a. Lateglacial and early Holocene palaeoclimatic reconstruction based on glacier fluctuations and equilibrium‐line altitudes at northern Folgefonna, Hardanger, western Norway. Journal of Quaternary Science 20, 179-198.

Bakke, J., Dahl, S.O., Paasche, Ø., Løvlie, R., Nesje, A., 2005b. Glacier fluctuations, equilibrium-line altitudes and palaeoclimate in Lyngen, northern Norway, during the Lateglacial and Holocene. The Holocene 15, 518-540.

Bakke, J., Dahl, S.O., Paasche, Ø., Riis Simonsen, J., Kvisvik, B., Bakke, K., Nesje, A., 2010. A complete record of Holocene glacier variability at Austre Okstindbreen, northern Norway: an integrated approach. Quaternary Science Reviews 29, 1246-1262.

Bakke, J., Nesje, A., Dahl, S.O., 2005c. Utilizing physical sediment variability in glacier-fed lakes for continuous glacier reconstructions during the Holocene, northern Folgefonna, western Norway. The Holocene 15, 161-176.

Bakke, J., Trachsel, M., Kvisvik, B.C., Nesje, A., Lyså, A., 2013. Numerical analyses of a multi-proxy data set from a distal glacier-fed lake, Sørsendalsvatn, western Norway. Quaternary Science Reviews 73, 182-195.

Bentley, M.J., Evans, D.J.A., Fogwill, C.J., Hansom, J.D., Sugden, D.E., Kubik, P.W., 2007. Glacial geomorphology and chronology of deglaciation, South Georgia, sub-Antarctic. Quaternary Sci Rev 26, 644-677.

Blaauw, M., 2010. Methods and code for ‘classical’ age-modelling of radiocarbon sequences. Quaternary Geochronology 5, 512-518.

Carr, S.J., Lukas, S., Mills, S.C., 2010. Glacier reconstruction and mass-balance modelling as a geomorphic and palaeoclimatic tool. EarthSurf.Proc.Land. 35, 1103–1115.

Clapperton, C., Sugden, D., Pelto, M., 1989a. Relationship of land terminating and fjord glaciers to Holocene climatic change, South Georgia, Antarctica, Glacier Fluctuations and Climatic Change. Springer, pp. 57-75.

Clapperton, C.M., 1971a. Geomorphology of the Stromness Bay-Cumberland Bay area, South Georgia.

Clapperton, C.M., 1971b. Geomorphology of the Stromness Bay - Cumberland Bay area, South Georgia. British Antarctic Survey Scientific Reports 70, 36.

Clapperton, C.M., 1990. Quaternary glaciations in the Southern Ocean and Antarctic peninsula area. Quaternary Science Reviews 9, 229-252.

Clapperton, C.M., Sugden, D., Birnie, R., Hansom, J., Thom, G., 1978. Glacier fluctuations in South Georgia and comparison with other island groups in the Scotia Sea. AA Balkema.

Clapperton, C.M., Sugden, D.E., 1988. Holocene glacier fluctuations in South America and Antarctica. Quaternary Science Reviews 7, 185-198.

Clapperton, C.M., Sugden, D.E., Birnie, J., Wilson, M.J., 1989b. Late-glacial and Holocene glacier fluctuations and environmental change on South Georgia, Southern Ocean. Quaternary Research 31, 210-228.

Cook, A.J., Vaughan, D.G., Luckman, A.J., Murray, T., 2014. A new Antarctic Peninsula glacier basin inventory and observed area changes since the 1940s. Antarct Sci 26, 614-624.

Croudace, I.W., Rindby, A., Rothwell, R.G., 2006. ITRAX: description and evaluation of a new multi-function X-ray core scanner. SPECIAL PUBLICATION-GEOLOGICAL SOCIETY OF LONDON 267, 51.

Dalziel, I.W.D., Dott, R.H.J., Winn, R.D.J., 1975. Tectonic Relations of South Georgia Island to the Southernmost Andes. Geological Society of America Bulletin 86, 1034-1040.

Gordon, J., 1987. Radiocarbon dates from Nordenskjold Glacier, South Georgia, and their implications for late Holocene glacier chronology. British Antarctic Survey Bulletin 76, 1-5.

Gordon, J.E., Hansom, J.D., 1986. Beach forms and changes associated with retreating glacier ice, South Georgia. Geografiska Annaler. Series A. Physical Geography, 15-24.

Gordon, J.E., Haynes, V.M., Hubbard, A., 2008. Recent glacier changes and climate trends on South Georgia. Global and Planetary Change 60, 72-84.

Gordon, J.E., Timmis, R.J., 1992. Glacier fluctuations on South Georgia during the 1970s and early 1980s. Antarctic Science 4, 215-226.

Heegaard, E., Birks, H.J.B., Telford, R.J., 2005. Relationships between calibrated ages and depth in stratigraphical sequences: an estimation procedure by mixed-effect regression. Holocene 15, 612-618.

Hodgson, D.A., Graham, A.G., Griffiths, H.J., Roberts, S.J., Cofaigh, C.Ó., Bentley, M.J., Evans, D.J., 2014a. Glacial history of sub-Antarctic South Georgia based on the submarine geomorphology of its fjords. Quaternary Science Reviews 89, 129-147.

Hodgson, D.A., Graham, A.G., Roberts, S.J., Bentley, M.J., Cofaigh, C.Ó., Verleyen, E., Vyverman, W., Jomelli, V., Favier, V., Brunstein, D., 2014b. Terrestrial and submarine evidence for the extent and timing of the Last Glacial Maximum and the onset of deglaciation on the maritime-Antarctic and sub-Antarctic islands. Quaternary Science Reviews 100, 137-158.

Lukas, S., 2012. Processes of annual moraine formation at a temperate alpine valley glacier: insights into glacier dynamics and climatic controls. Boreas 41, 463-480.

Nesje, A., 1992. A piston corer for lacustrine and marine sediments. Arctic and Alpine Research, 257-259.

Oppedal, L., Bakke, J., Paasche, Ø., Werner, J., van der Bilt, G.W., 2018. Cirque Glacier on South Georgia Shows Centennial Variability over the Last 7000 Years. Front. Earth Sci.

Porter, S.C., 1975. Equilibrium line altitudes of late quaternary glaciers in the Southern Alps, New Zealand. Quaterary Research 5, 27–47.

Rosqvist, G.C., Schuber, P., 2003. Millennial-scale climate changes on South Georgia, Southern Ocean. Quaternary Res 59, 470-475.

Smith, J., 1960. Glacier problems in South Georgia. Journal of Glaciology 3, 707-714.

Stone, B.S., Ph.D., 1980a. The geology og South Georgia: IV. Barff Peninsular and Royal Bay areas, in: Survey, B.A. (Ed.). Earth Science Division, British Anartic Survey and Department of Geologiacal Science, University of Birmingham, Cambridge.

Stone, P., 1980b. The geology of South Georgia: IV. Barff Peninsula and Royal Bay areas. British Antarctic Survey, Cambridge.

Trendall, B.S., Ph.D., 1953. The Geology of South Georgia - 1, in: Survey, F.I.D. (Ed.). Falkland Island Dependencies Scientific Bureau, London.

Trendall, B.S., Ph.D., 1959. The Geology of South Georgia - 2, in: Survey, F.I.d. (Ed.). Falkland Island Dependencies Scientific Bureau, London.

Van der Bilt, W., Bakke, J., Werner, J., Paasche, Ø., Rosqvist, G., Vatle, S., 2017. Late Holocene glacier reconstruction reveals retreat behind present limits and two-stage Little Ice Age on subantarctic South Georgia. Journal of Quaternary Science 32, 888–901.
